# Multi-omics reveals the positive leverage of plant secondary metabolites on the gut microbiota in a non-model mammal

**DOI:** 10.1186/s40168-021-01142-6

**Published:** 2021-09-22

**Authors:** Le Wang, Guangping Huang, Rong Hou, Dunwu Qi, Qi Wu, Yonggang Nie, Zhenqiang Zuo, Rui Ma, Wenliang Zhou, Yingjie Ma, Yibo Hu, Zhisong Yang, Li Yan, Fuwen Wei

**Affiliations:** 1 Key Laboratory of Animal Ecology and Conservation Biology, Institute of Zoology, Chinese Academy of Sciences, Beijing, 100101 China; 2 University of Chinese Academy of Sciences, Beijing, 100049 China; 3 Chengdu Research Base of Giant Panda Breeding, Chengdu, 610081 China; 5 Beijing Institutes of Life Science, Chinese Academy of Sciences, 100101 Beijing, China; 4 Center for Evolution and Conservation Biology, Southern Marine Science and Engineering Guangdong Laboratory (Guangzhou), 511458 Guangzhou, China; 6 Sichuan Academy of Giant Panda, Chengdu, 610081 China

**Keywords:** PSMs, Flavonoids, Giant pandas, Metabolomics, Metagenomics

## Abstract

**Background:**

Flavonoids are important plant secondary metabolites (PSMs) that have been widely used for their health-promoting effects. However, little is known about overall flavonoid metabolism and the interactive effects between flavonoids and the gut microbiota. The flavonoid-rich bamboo and the giant panda provide an ideal system to bridge this gap.

**Results:**

Here, integrating metabolomic and metagenomic approaches, and in vitro culture experiment, we identified 97 flavonoids in bamboo and most of them have not been identified previously; the utilization of more than 70% flavonoid monomers was attributed to gut microbiota; the variation of flavonoid in bamboo leaves and shoots shaped the seasonal microbial fluctuation. The greater the flavonoid content in the diet was, the lower microbial diversity and virulence factor, but the more cellulose-degrading species.

**Conclusions:**

Our study shows an unprecedented landscape of beneficial PSMs in a non-model mammal and reveals that PSMs remodel the gut microbiota conferring host adaptation to diet transition in an ecological context, providing a novel insight into host-microbe interaction.

**Video abstract**

**Supplementary Information:**

The online version contains supplementary material available at 10.1186/s40168-021-01142-6.

## Introduction

Flavonoids are ubiquitous plant secondary metabolites (PSMs), a class of polyphenols comprising diverse subclasses [1]. Unlike macronutrients, they are nutritionally nonessential but explicitly functional phytochemicals [1]. Based on a considerable number of epidemiological studies, flavonoid consumption is evidently associated with several beneficial health effects, e.g., improvement of metabolic parameters in obesity and interventions for weight loss [2], immune response enhancement [3], and amelioration of metabolic syndrome [4]. All of these health-promoting effects are mediated by the gut microbiota as a whole [2–4]. As the most typical PSMs, flavonoids in bamboos are of high content, and therefore, bamboo leaves have been applied in the food industry and traditional medicine [5]. One flavonoid-rich bamboo leaf extract has multiple biological effects, such as antibacterial, antiviral, and anti-inflammatory effects, and it has been used as a pharmaceutical intermediate, dietary supplement and food additive [5]. However, few flavonoids in bamboo leaves, such as orientin and vitexin, are well-known and used as nutraceuticals [6, 7], the overview of flavonoid profiles has not been well studied. Previous studies are limited by classic chromatographic separation technology, resulting in a lack of systemic research on bamboo flavonoids. Furthermore, studies on model animals cannot directly infer their interactive effects with the gut microbiota of non-model mammals, especially in the case of wildlife with habitual intake of flavonoids.

The giant panda (*Ailuropoda melanoleuca*) is the most famous bamboo specialist belonging to the order Carnivora. It possesses the general characteristics of carnivores. For example, its digestive tract is characterized by short overall length, long and dense villi, and a large number of secretory cells [8]. Moreover, it has its own specific characteristics in terms of morphology, ecology, physiology, and genetics for adaptation to the specialized bamboo diet [9]. To maximize nutritional and energetic intake, the giant panda consumes a large amount of food per day (10–18 kg of leaves or stems or approximately 38 kg of shoots) [10]. In addition to the panda’s own phenotypic strategies, its gut microbiota also plays a key role in this evolutionary process of dietary adaptation [11–16]. Comparative metagenomics has revealed that the composition of the giant panda gut microbiome is simple with low species diversity [17]. The gut microbiota has been proven to be of great functional importance, especially in the nutritional utilization of bamboo [11–14]. For example, microbial cellulases play an irreplaceable role in cellulose decomposition [11], and the microbiome harbors a complete pathway for vitamin synthesis [16]. However, few studies have uncovered the effect of habitual flavonoid intake on the gut microbial configuration and the microbial effectiveness in the degradation of flavonoids. The specialized bamboo diet along with the indispensable function of the gut microbiota of the giant panda makes it an ideal model for studying habitual dietary flavonoid metabolism in vivo and the coordinated effects of flavonoids and the gut microbiome on host health.

In this study, we systematically screened flavonoids in the diet, feces, and plasma by a widely targeted metabolomic analysis. Both wild and captive giant pandas were included. For the wild population, diets and feces were collected by direct tracking in the Foping National Natural Reserve, Shaanxi, Qinling Mountains. For the captive population, diets and feces, as well as plasma, were sampled in the Chengdu Research Base of Giant Panda Breeding. Based on the sampling sites and seasonal diets, samples were divided into four groups (Table S1): WL (wild pandas with leaves of the bamboo *Bashania fargesii* as diet), WS (wild pandas with shoots of the bamboo *B. fargesii* as diet), CL (pandas in captivity fed on the leaves of *B. fargesii*), and CS (pandas in captivity fed on shoots of the bamboos *Pleidolastus amarus* and *Phyllostachys nidularia,* corresponding to subclass CS_I and CS_II, respectively).

Compared to the context of dietary flavonoids in bamboo leaves and shoots, the targeted metabolome in plasma characterizes direct absorption by the host giant panda, whereas fecal flavonoids represent the final metabolic outcomes, indicating comprehensive bioutilization processes. To uncover the bidirectional biological interactions between flavonoid availability and the gut microbiota, we characterized the metagenome in real-time by shotgun sequencing, established the potential links of flavonoids with the global gut microbiota configuration and explored the possible microbial pathways involved in the conversion of flavonoids.

## Results

### Substantial changes in flavonoid profiles in bamboo leaves and shoots show a marked variation between seasonal diets

**Fig. 1:**
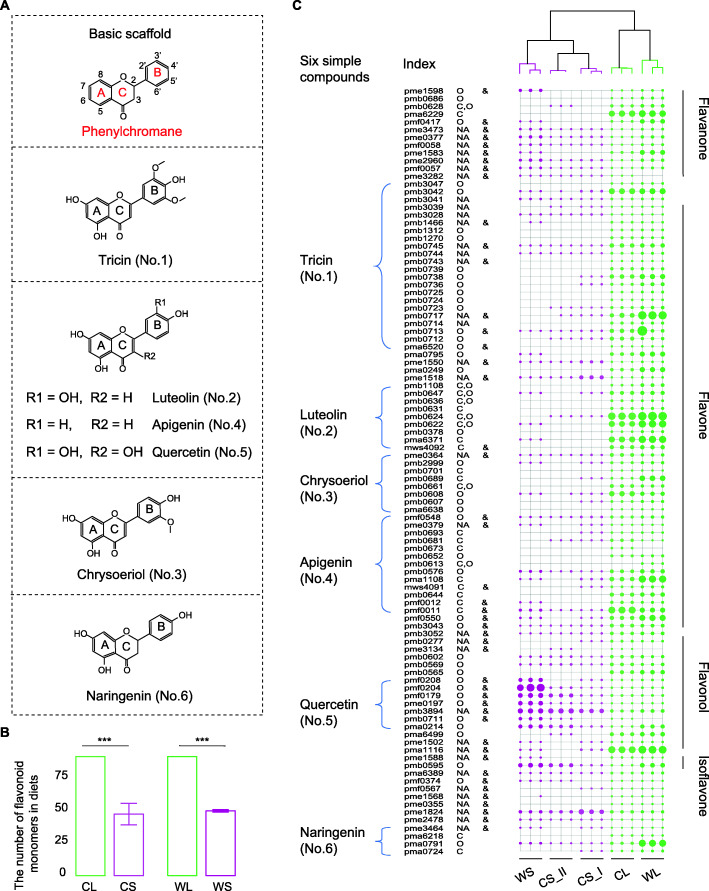
Structural features and distribution of flavonoids in seasonal diets. **A** Basic scaffold of flavonoids and representative aglycone monomers detected in dietary bamboos of giant pandas. Flavonoids are constituted of two aromatic rings (**A**, **B**) linked through three carbons that usually form an oxygenated heterocycle (C ring) (a C6-C3-C6 skeleton named phenylchromane). This C ring is characteristic of each flavonoid subfamily. The basic structure of the individual flavonoids is modified by hydroxy, methoxy and alkyl groups attached to the A- and B-rings. For each representative aglycone with numbers in parentheses, the structural formula and the common name are indicated. **B** Comparison of the diversity of flavonoid monomers in bamboo leaves versus shoots. **C** Comparison of the abundance of flavonoids in bamboo leaves versus shoots. The unrooted tree on the left shows the hierarchical clustering among diet samples by hierarchical clustering based on the Bray-Curtis distance. The bubble chart on the right shows the distribution of all flavonoids. The sizes and colors of the circles indicate the abundance and food type in different seasons, respectively. The absence of a bubble at one node indicated no flavonoid was detected in the corresponding sample. The subjects of indexes are listed in Table S2. Ampersand denotes flavonoids with precise structural identification. Most flavonoids were identified as in the glycoside forms. C, C-glycoside; O, O-glycoside; C, O, C, O-di-glycoside; NA, no glycosyl. For **B** and **C**, four groups represented samples of two types of bamboo diets (leaves and shoots) from two populations of giant pandas (wild and captive ones): WL, wild pandas with leaves of the bamboo *B. fargesii* as diet; WS, wild pandas with shoots of the bamboo *B. fargesii* as diet; CL, pandas in captivity fed on the leaves of *B. fargesii*; CS, pandas in captivity fed on shoots of the bamboos *P. amarus* and *P. nidularia*, corresponding to subclass CS_I and CS_II, respectively. The green and purple colors represent the bamboo leaf and shoot individually. And both the group information and colors were the same as in Figs. 2 and 3

In total, 15 samples representing five types of diets (one kind for each type of three mixed duplicates) (Table S1) were used for flavonoids determination, according to the methods of widely targeted metabolome based on liquid chromatography-mass spectrometry (LC-MS) [18]. Based on the local database, 97 specific flavonoid monomers were identified in the bamboo samples, including 62 flavones, 18 flavonols, 12 flavanones, 3 isoflavones, 1 chalcone, and 1 flavanonol, most of which were in glycoside form (Fig. 1A–C; Table S2). We found that most of the flavonoids identified here were derived from six simple compounds, including tricin, apigenin, luteolin, chrysoeriol, quercetin, and naringenin (Fig. 1C). We also detected 2 polyphenols (protocatechuic acid and protocatechuic aldehyde), which were also included in the subsequent analysis due to their small number, similar polyphenolic structures, and bioactivities with flavonoids. In a previous study, a limited number of flavonoid compounds in bamboo were identified or experimentally tested, including (iso)orientin and (iso)vitexin, which are flavone C-glucosides of luteolin and apigenin, respectively [19].

Comparative analyses showed that bamboo leaves contained more diverse flavonoids than shoots (Wilcoxon rank sum test, *P* < 0.05) (Fig. 1B). There were approximately 100 flavonoid monomers in bamboo leaves (WL, 95; CL, 97), which greatly outnumbered those in bamboo shoots (WS, 52; CS_I, 57; CS_II, 40) (Fig. 1B). Only two monomers (one flavonol: quercetin 7-O-rutinoside; one flavanone: afzelechin) were discovered in wild shoots, and these were not detected in wild leaves. Overall, substantial differences between bamboo leaves and shoots were identified in our study, indicating a marked variation of flavonoid ingestion of giant pandas across the year.

### Systematic metabolic snapshots indicate considerable contributions of the gut microbiota to the utilization of flavonoids

**Fig. 2:**
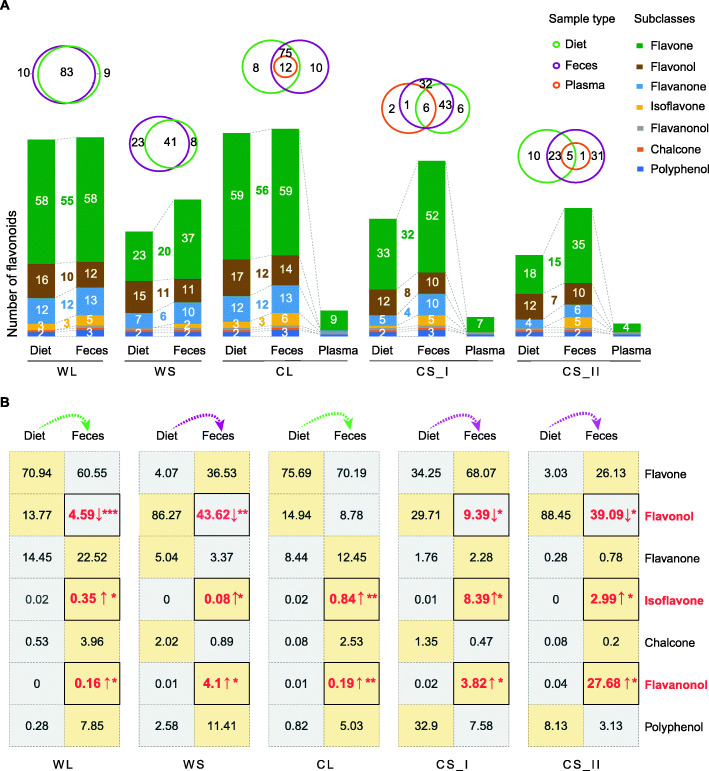
The global distribution of flavonoids in the diet, feces and plasma of giant pandas and changes in the proportions of subclasses in diet and feces. **A** Top, Venn diagrams of flavonoids in three sample types. Below, bar plots of flavonoids at the subclass level. Each figure on the bars represents the number of flavonoids in the subclass detected in the corresponding samples. No figures are given when the numbers were less than three. The numbers between diets and feces are the numbers of overlapping flavonoids within each subclass. **B** Figures on the bars represent the percentages of seven subclasses. Upwards arrow and downwards arrow indicate the percentage in feces was higher and lower than in diet respectively. ***P < 0.001, **P < 0.01, *P < 0.05

To identify the absorption, distribution, and bioconversion of flavonoids within giant pandas, we scanned plasma samples from captive individuals (*n* = 44) with the same methods of LC-MS and specific preliminary treatment in extraction as described in “Materials and methods”. Only a fraction of the parent flavonoids entered the bloodstream for captive group (on leaves in CL, 12 monomers; on shoots in CS_I, 6 monomers; and on CS_II, 6 monomers) (Table S3). Most of them were flavones (e.g., tricin, nobiletin and tangeretin) and flavone glycosylated derivatives (e.g., orientin from luteolin and schaftoside from apigenin) (Fig. 2A; Table S2).

Next, we examined the seasonal flavonoid profiles of feces (for wild groups, WL: *n* = 12, WS: *n* = 10; for captive groups, CL: *n* = 28, CS_I: *n* = 11; CS_II, 10). For these dietary flavonoids, more than 70% of the monomers were detected again in feces, further suggesting their low absorbability in giant pandas, consistent with the low detection rate in plasma. But some other flavonoids were undetectable in feces (for wild groups, WL, 9 monomers, WS, 8 monomers; for captive groups, CL, 8 monomers; CS_I, 6 monomers; CS_II, 10 monomers), indicating the full utilization of partial dietary flavonoids (Fig. 2A). Among all the flavonoids detected in feces, approximately 10% and more than 30% new flavonoids were present in the leaf eating (WL, 10 out of 93; CL, 10 out of 97) and shoot eating seasons (WS, 23 out of 64; CS_I, 33 out of 82; CS_II, 32 out of 60), respectively (Fig. 2A). Therefore, these results showed a coherent view of the absorption and bioconversion of flavonoids within the giant panda, as follows. Due to the short retention time of the digesta in the gastrointestinal tract (5 to 11 h) [20], the high detection rates of dietary flavonoids in feces in native forms suggested that the apparent digestibility of dietary flavonoids is low. The low finding rates in plasma indicated that the majority of dietary flavonoids could not enter the bloodstream to produce global physiological effects in giant pandas through the circulation. However, the other monomers that were completely metabolized or newly generated confirmed the extensive utilization and biotransformation of flavonoids by the gut microbiota, in which the flavonoids would exert local effects in the gut by the mutual interaction with symbiotic bacteria.

We then identified the flavonoid utilization preferences in giant pandas based on the changes of each subclass/monomer in proportion between diets and corresponding feces (see “Materials and methods” for details). At the flavonoid subclass level, the relative proportion of flavonols in feces was markedly lower than that in diets (*P* < 0.05), in contrast to those of isoflavones and flavanonols (Fig. 2B). Thus, flavonols showed greater absorbability and utilization than other subclasses. Among specific compounds, we identified a total of 38 monomers with high absorbability by the same comparative analysis between diets and feces (Table S4). Of these 38 monomers, 27 were in glycosylated form, such as narcissoside (3′-methylquercetin 3-O-rutinoside), apigenin 6,8-C-diglucoside, and hyperoside (quercetin 3-D-galactoside). Three flavonol monomers present in the diets were undetectable in the feces of all groups, including two derivatives of quercetin (di-O-methylquercetin and quercetin 3-O-rutinoside) and one O-glycoside of syringetin. These three were also absent in plasma. Combined with the changes in the proportions of subclasses, these results indicated that although flavonols were more easily absorbed, they did not enter circulation. Together, these results characterized the preference ordering of flavonoid utilization by the giant panda. The flavonols are the highest; secondly flavone, flavanone, and chalcone; and successively, isoflavone and flavanonol were the lowest. Besides, we infer that flavonols were mainly utilized and biotransformed by the gut microbiota.

### Associations of flavonoids with gut microbial species

**Fig. 3:**
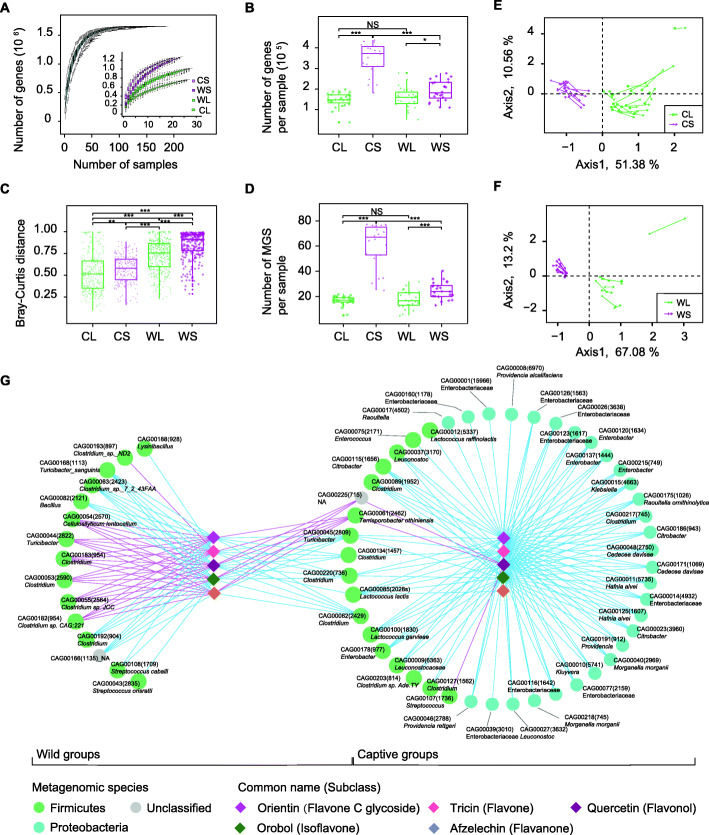
Seasonal gut microbiome variations and associations between the fecal metabolome and metagenome. Comparison between shotgun sequencing data of stool samples from the leaf season (WL, *n* = 28; CL, *n* = 27) and shoot season (WS, *n* = 24; CS, *n* = 21). **A** Rarefaction curves based on gene count. **B** Box plot of the gene count in four groups. **C** β-Diversity (Bray-Curtis similarity index) of the four groups at the gene level. **D** Box plot of the number of metagenomic species (MGS) in the four groups. For **B**, **C** and **D**, a two-tailed Wilcoxon rank-sum test was used to determine significance. *P value < 0.05, ***P value < 0.001. NS, not significant. Boxes represent the interquartile ranges (IQRs) between the first and third quartiles, and the line inside the box represents the median; whiskers represent the lowest or highest values within 1.5 times the IQR from the first or third quartiles. Circles represent data points beyond the whiskers. A plot of the first two axes (Axis1 and Axis2) of a CoIA of fecal metabolites and all CAGs of captive (**E**) and wild samples (**F**). Covariation showed concordance of seasonal structural shifts between fecal metabolites and all CAGs. Each sample is represented with an arrow. Green and magenta colors represent the leaf eating season and shoot eating season, respectively. The sample projections in the metabolome and metagenome space are represented by the starting point and the end of the arrow, respectively. The length of the arrow is inversely proportionally to the consensus between these two datasets. **G** Correlation network between fecal metabolites (five monomers representing five subclasses of flavonoids) and the metagenomic species (MGS) that were different between seasons. For clarity, only significant strong correlations (Spearman’s rank correlation: |*r*| > 0.6, *P* < 0.05) were considered. Red and blue edges denote Spearman’s rank correlation coefficient *r* > 0.6 and r < -0.6, respectively. The numbers in parentheses next to each species name represent the number of genes in the MGS

Metagenomic data for 84 new fecal samples of giant pandas (26 samples from wild pandas and 58 samples from captive pandas) together with 105 published data sets [13–15, 21] were integrated (Table S5), among which 100 stool samples were included in four groups based on sampling sites and diet composition (WL, *n* = 28; WS, *n* = 24; CL, *n* = 27; CS, *n* = 21). In total, a catalog of 32,701,194 nonredundant microbial genes was established based on 1730.64 gigabases (Gb) of paired-end read sequence data, with an average of 9.16 (± 4.74) Gb for each sample. The two leaf diets had markedly lower gene richness (the number of genes per sample) than the shoot diets (WL vs. WS, CL vs. CS) (Fig. 3A, B). The gene richness of the CS group was much higher than that of the WS group (*P* < 0.01, Wilcoxon rank sum test, false discovery rate (FDR) < 0.05), but it dropped markedly in the CL group, in which it was not significantly different from that of the WL group (Wilcoxon rank sum test, FDR > 0.05). In addition, we observed higher β diversity (Bray-Curtis distances) in the shoot eating seasons than in the leaf eating seasons (Fig. 3C), indicating a more heterogeneous community structure in the shoot eating season. Using a metagenome-wide association analysis, we identified 12.77% of genes in the wild groups (743,853 genes in total) and 40.13% of genes in the captive groups (884,685 genes in total) associated with seasonal diet transition (*P* < 0.01, Wilcoxon rank sum test, FDR < 0.05). Consistent with this result, the Bray-Curtis distances of different genes showed significant alterations from shoot diet to leaf diet in both wild and captive groups (Figure S1).

To explore the biological diversity of gut microbial systems at the level of biological entities, we used a method basing on binning co-abundant genes (CAG) across a series of metagenomic samples [22]. A total of 144 metagenomic species (MGS) containing > 700 genes (Table S6) were identified to represent the genomes of the ecological bacterial community [22]. The wild groups contained 61 MGS dominated by *Firmicutes* (WL, 45; WS, 54), and the captive groups had 101 MGS dominated by *Proteobacteria* and *Firmicutes* (CL, 31; CS, 96). Although the gut microbiota of the wild and captive populations contained distinct taxa (Figure S2), they shared the same change trends from the shoot eating to leaf eating stages. Consistent with the results at the gene level, we found that the numbers of MGS in WL and CL were markedly lower than those in WS and CS (*P* < 0.01, Wilcoxon rank sum test, FDR < 0.05) (Fig. 3D; Figure S3). Moreover, the seasonal variation in MGS richness in the wild was magnified in captivity. Seasonal differential MGS in the wild and captivity accounted for 37.70% (23 out of 61) and 77.23% (78 out of 101) of the total number of MGS, respectively (Figure S4).

We then established comprehensive associations between the metagenome and metabolome. Overall, co-inertia analysis (CoIA) showed a close association of metabolites with the altered microbial species (Fig. 3E, F). In total, flavonoids showed more significant seasonal variation (Figures S5-S6) and more extensive links with MGS than non-flavonoid compounds (NFCs) (Figures S7-S8). Flavonoids selectively promote specific commensals at a higher level of classification, such as phylum (Fig. 3G). For captive giant pandas, a sizeable proportion of microbial commensals belonging to *Proteobacteria* may have less flavonoid conversion potential. As a result, the more the flavonoid intake in the leaf season is, the more the number of negative responses offered by gut microbial residents. Nevertheless, the majority of the gut microbiota of wild pandas were members of *Firmicutes*, which responded more positively to the abundant flavonoid intake in leaf eating seasons (Figures S7-S8).

### Dietary flavonoids induce the seasonal dynamics of gut microbiota

Although the gut microbiota of captive giant pandas was very different from that of wild population for multifactorial effects, it showed higher diversity in shoot eating stage at the level of gene richness and the metagenomic species (MGS) for both the wild and captive population (Fig. 3B–D). We suggest that except for the differences between wild and man-made environments, there are key factors in dietary bamboo leaves driving the extensive scavenging of bacterial species. Based on extensive links of flavonoids with gut microbial species, we used fecal bacteria culture in vitro to test impacts of flavonoids.

**Fig. 4:**
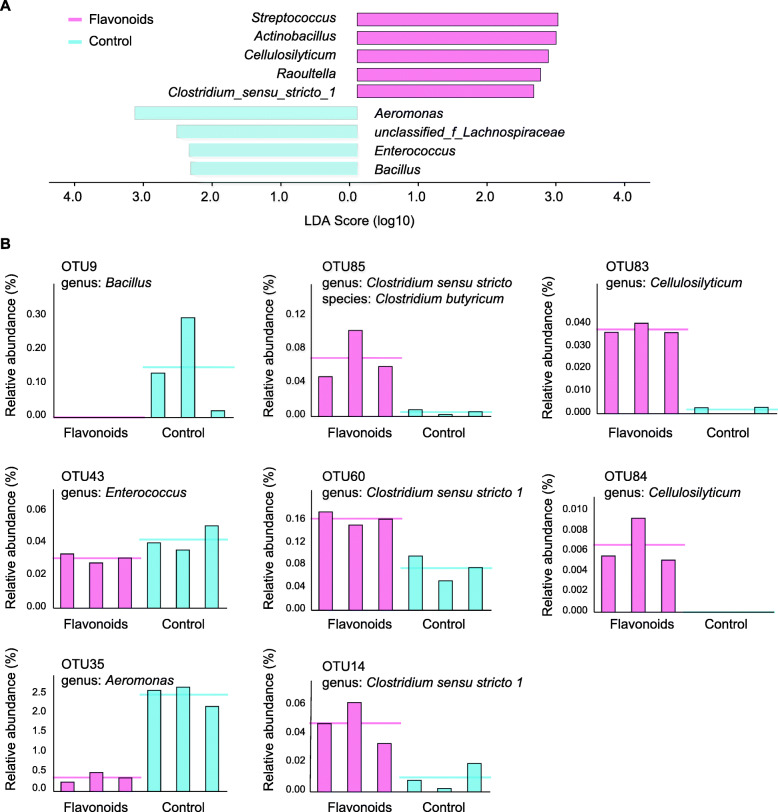
The composition variation of fecal bacteria in vitro by flavonoids supplementation. **A** Bacterial differences between the treatment (flavonoids) and control groups at the genus level (linear discriminant analysis (LDA) score > 2). **B** Differentially represented OTUs between the flavonoids and control groups

Flavonoid-induced microbial variation in vitro evidenced the effects of flavonoids on the composition of gut microbiota and confirmed associations between flavonoids and MGS at the omics level (Fig. 4A, B). For example, compared with the shoot eating stage, the *Clostridium sensu stricto 1* in leaf eating stage has higher abundance for wild pandas [13]. Here, three OTUs belonging to *Clostridium sensu stricto 1* (including OTU85, OTU60 and OTU14) were enriched in the flavonoid group. In addition, two OTUs belonging to *Cellulosilyticum* (including OTU83 and OTU84) were also enriched by the additive flavonoid. Both *Clostridium sensu stricto 1* and *Cellulosilyticum* belong to *Firmicutes*. For the omics-based association, culture in vitro also showed consistent changes by flavonoid supplement. For example, CAG00054 belonging to *Cellulosilyticum* showed positive associations with flavonoids. In contrast to the change of *Cellulosilyticum*, *Bacillus* (OTU9) and *Enterococcus* (OTU35) were inhibited by the addition of flavonoids. Associative analyses also found that CAG00082 (*Bacillus*) showed negative associations with flavonoids (Fig. 4B). Together, these data indicate the substantial effect of bamboo flavonoids in shaping the seasonal composition of gut microbiota.

### The fecal microbiome possesses enzymes involved in flavonoid bioconversion

**Fig. 5:**
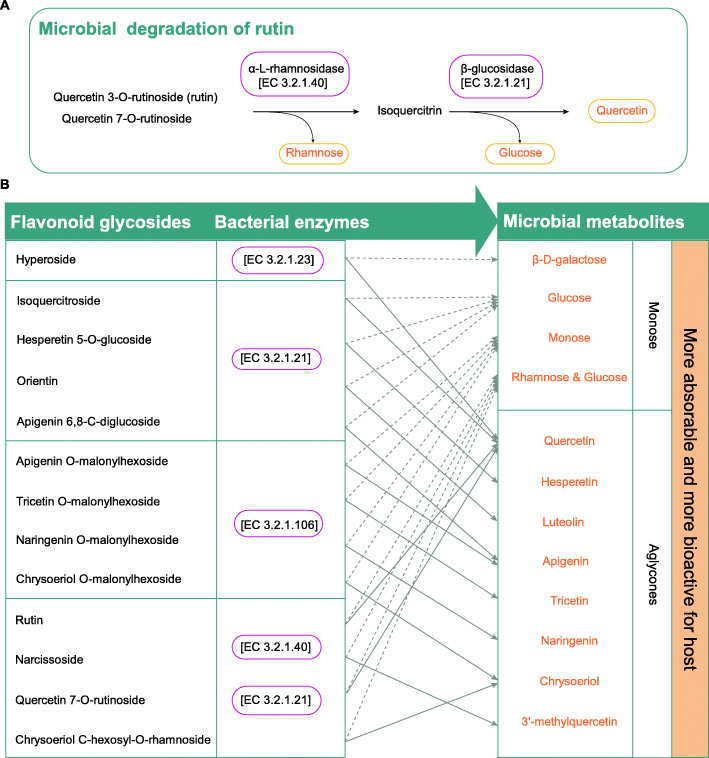
Gut microbial pathway involved in flavonoid conversion. **A** Microbial conversion of rutin by gut microbial enzymes. **B** Deglycosylation of flavonoids by microbial glycoside hydrolases. Dietary flavonoids with different glycosyls and their related glycoside hydrolases are separated into four boxes. Dotted and solid lines indicate the monose and the aglycones respectively

With the evidence-based effects of flavonoids on the composition of gut microbiota, we suspect that the gut bacteria may contribute to the utilization preference and biotransformation of flavonoids in the giant panda. All genes were annotated against the Kyoto Encyclopedia of Genes and Genomes (KEGG) database to clarify microbial functions in the utilization and conversion of flavonoids. We first concentrated on those monomers with full utilization (see above and Fig. 2A). We observed that rutin was present in both bamboo leaves and shoots, but it was consistently absent in blood and feces. Rutin is a flavonol glycoside composed of quercetin and rutinose, a disaccharide of rhamnose and glucose (Fig. 5A). We found two enzymes in samples of all groups, namely, α-L-rhamnosidase [EC 3.2.1.40] and β-glucosidase [EC 3.2.1.21], which are involved in the two key steps of rutin conversion [23].The α-L-rhamnosidase specifically cleaves terminal α-L-rhamnose, followed by β-glucosidase cleaving glucose (Fig. 5A). Thus, the high utilization of rutin which was close to completeness was due in large part to the microbial enzymes. These enzymes also catalyzed the full degradation of quercetin 7-O-rutinoside in the CL, WS, and CS_II groups.

In addition to the above two enzymes, other glycoside hydrolases were also present in the gut microbiota of giant pandas, including α-galactosidase [EC 3.2.1.22], β-galactosidase [EC 3.2.1.23], α-glucosidase [EC 3.2.1.20], and mannosyl-oligosaccharide glucosidase [EC 3.2.1.106]. These enzymes might contribute to the high utilization of 27 glycosyl flavonoids and result in marked decreases in their percentages from diets to feces. With extensive microbial involvement, dietary flavonoids were deglycosylated to aglcones and monose compounds (Fig. 5B). For instance, hyperoside (quercetin 3-D-galactoside) could be hydrolyzed by β-galactosidase with the release of galactose (Fig. 5B).

### The functional readout of seasonal gut microbiota shows that flavonoids reduce microbial virulence factors

**Fig. 6:**
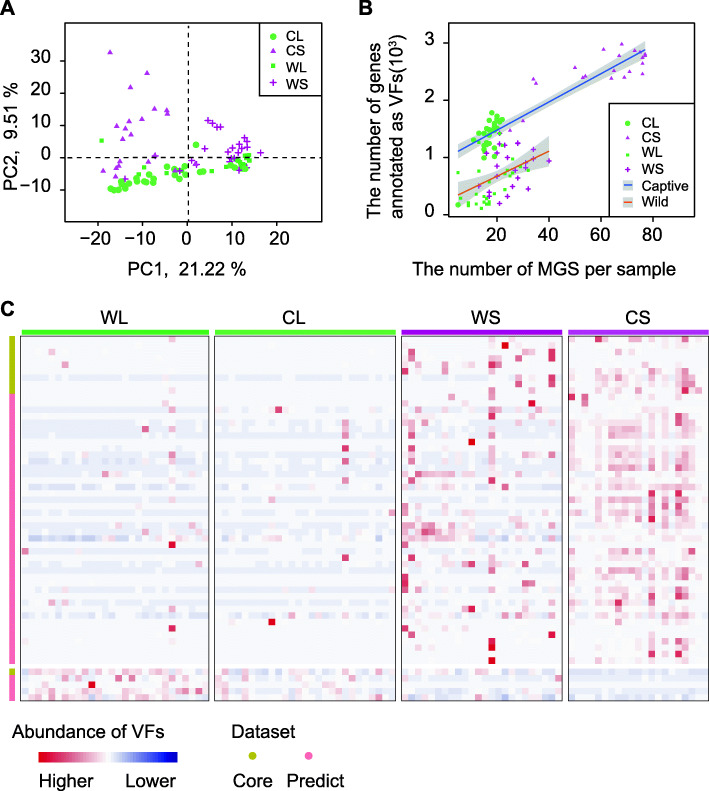
The seasonal variations of microbial virulence factors. **A** Distribution of seasonally differential genes involved in QS. The numbers of genes with higher abundance in the leaf eating season (WL and CL) or shoot eating season (WS and CS) are shown. **B** PCA plot based on all virulence factor (VF)-related genes. **C** The positive associations of gene richness involving VFs and MGS number per sample. MGS, metagenomic species. A linear fitting (the solid lines in two colors and the 95% confidence interval in gray) of the number of VF-related genes and the MGS number per sample showed the covariations (wild groups: *P* = 0.0013**, captive groups: *P* < 2e− 16***, **P value < 0.01, ***P value < 0.001). **D** Heatmap of different VFs with seasons. For clarity, only differential VFs shared by wild and captive samples are shown

As certain flavonoids have been proven to inhibit virulence factors (VFs) of enteropathogenic bacteria via direct antibacterial effects [24] and interfere with “quorum sensing” (QS) [25, 26], we further determined whether the homeostasis of the gut microbial ecosystem was fluctuated with seasons following the seasonal microbial composition under the driving force of dietary flavonoids. We focused on the microbial VFs and VF-related QS pathway. A total of 194 KEGG orthologs (KOs) involving the QS pathway were identified. Most of the seasonally differential KOs (19 of 25 KOs in wild groups and 57 of 65 KOs in captive groups) had much higher abundance in the shoot eating season than in the leaf eating season (*P* < 0.01, Wilcoxon rank sum test, FDR < 0.05) (Fig. 6A). For example, the fucose-sensing system FusKR is a two-component signal transduction system that is required for the colonization of the pathogen enterohaemorrhagic *Escherichia coli* (EHEC) and modulates the virulence gene expression of EHEC [27]. Here, both the histidine sensor kinase (FusK) and the response regulator (FusR) showed higher abundance in the shoot eating season (Figure S9, K20263 and K20264, respectively). BapA (K20276), a large secreted protein required for biofilm formation, contributes to both biofilm formation and invasion by pathogenic *Salmonella* [28]. Thus, the higher abundance of QS-related genes in the shoot eating season indicated that there were more pathogenic bacteria in the gut microenvironment when the pandas fed on bamboo shoots.

Next, the nonredundant gene catalog was annotated against the virulence factor database (VFDB, http://www.mgc.ac.cn/VFs/). A total of 4501 genes were annotated as 647 VFs (including 186 from the core dataset and 461 from the predicted dataset), which distinguished seasonal differences among all samples fairly well (Fig. 6B). Significantly more VF-related genes were detected in samples from captive pandas than in samples from wild pandas (Fig. 6C; Figure S10A). Ninety-six and 299 seasonally differential VFs were obtained in wild and captive settings, respectively (*P* < 0.01, Wilcoxon rank sum test, FDR < 0.05). Consistent with the KO abundance in the QS pathway, nine-tenths of the VFs were much more abundant in the shoot eating season than in the leaf eating season (Fig. 6D; Figure S10B, C). In addition, the number of VF-associated genes was markedly associated with MGS richness both in the wild (*P* = 0.0013) and in captivity (*P* < 2e− 16) (Fig. 6C). Together, the higher flavonoid intake in the leaf eating season may confer health benefits in the gut for the hosts by regulating the composition of gut microbiota, for which the microbial VFs were downregulated in the whole gut microbial community.

## Discussion

The giant panda is a flagship species for biodiversity conservation. Here, taking advantage of metabolomic and metagenomic approaches, we have extended the breadth and depth of our knowledge regarding the nutritional ecology of giant pandas by unraveling the interaction between PSMs and gut microbiome. The widely targeted metabolomic analyses showed diverse flavonoid monomers in the dietary bamboos of giant pandas in both the wild and captive settings, suggesting that giant pandas ingest a higher variety/number of flavonoids than previously known [29].

This is the first study to quantify the relative abundance of beneficial PSMs in the bloodstream of giant pandas. Unlike humans and other laboratory animal models, the giant panda has unique feeding behaviors. It spends half its time feeding daily and ingests a comparable amount of bamboo during the night [8]. Based on these specific daily activity rhythms, our data reflected the normal presence of flavonoids in the circulatory system. The eleven monomers entering the blood circulation may reach their target organ and exert a direct influence on giant pandas [30]. For example, orientin, nobiletin, and tangeretin have cardiovascular-protective effects. Orientin could protect vascular barrier integrity in mice by inhibiting hyperpermeability, the expression of cell adhesion molecules, and the adhesion and migration of leukocytes [31]. Both nobiletin and tangeretin are potential antithrombotic agents that inhibit platelet function based on the experimental tests on mice and human [32, 33]. We suspect that these beneficial effects are of great importance for health maintenance in giant pandas. Although it is now difficult to validate the medicinal roles of flavonoids in the physiological maintenance of giant pandas, the mechanism of action of dietary flavonoids on the cardiovascular system of mammals deserves further research.

In addition to the documented effect of gut microbiota on giant pandas, the targeted flavonoid profiles in feces show that flavonoids are bound to undergo extensive microbial degradation and bioconversion. The microbial contribution to the metabolism and bioconversion of flavonoids is evidenced jointly by the fecal metabolome and fecal metagenome. At the metabolome level, among all the dietary flavonoids from bamboos, different subclasses or even specific monomers had different degrees of absorbability and bioconversion. Second, partial dietary flavonoids were undetectable in feces and newly generated monomers were present in feces. For example, flavonols showed higher usage than other subclasses; however, no specific monomer was present in blood. Rutin, the most widespread glycosidic form of quercetin, was present in the diets of both wild and captive pandas. However, it was fully metabolized and was therefore undetectable in the feces of all five groups, and its aglycone was generated. At the metagenome level, key enzymes involved in the hydrolysis of glycosyl flavonoids were also present. For example, α-L-rhamnosidase and β-glucosidase both play an important role in the metabolism and pharmacological effect of rhamnoglycosides [23]. Factually, it is well documented that rutin has intestinal anti-inflammatory effects in experimental models of rat colitis and theses effects are largely dependent on the quercetin (its aglycone) release [34]. That is, compared to the glycosylated forms, the biological activities of quercetin are more effective [34]. According to this principle, we propose that the gut microbiota contribute to the preference utilization of dietary rutin in giant pandas, conferring host maximum extract of beneficial elements in diets. Therefore, flavonoids not only provide substrates (carbon sources) for the gut microbiota but also provide microbial products that are more easily absorbed by hosts with more effective bioactivities, such as aglycones and monose.

Up to now, it is complicated to uncover the formation mechanism of the gut microbiota of diverse mammals, which involve many ecological and environmental factors, as well as hosts themselves [35, 36]. In the present study, the gut microbial diversity of captive population is much higher than the wild one. We suspect the captive surroundings are totally different from the wild (such as the altitude, temperature, food resources, especially the human activities), which are much more complex than the wild. Besides, the bamboos (both shoots and leaves) in captive settings are not as fresh as the wild diets, because they are always transported to the base at least one day in advance, during which time many bacteria were transmitted to diets from man-made environment.

Diets are one of the well-known factors to drive changes in both gut microbiome composition and their functions both on the interspecific/ phylogenetic scales [16, 35–37] and on the interspecific scales [13, 38–40]. For example, the bamboo-eating giant panda harbors a carnivore-like gut microbiota, which is distinctly different from that of other mammalian herbivores with low species diversity [17, 41]. In the meanwhile, its gut microbiota shows distinct diet-associated seasonal variations [13, 41]. Studies addressing how the microbiota of wild animals responds to dietary elements and how these animals achieve optimal utilization of diets are highly warranted [42]. Due to their reclusive nature and the difficulty of obtaining biological samples of giant pandas, to date, there are limited metagenomic data on the giant panda and no specific food elements have been linked to the longitudinal dynamics of gut microbial communities or even specific bacterial taxa. Dietary flavonoids are very relevant to microbial community assembly [43]. Here, based on the results of metabolome, fecal bacteria culture in vitro with flavonoids evidenced that the seasonal differential intakes of flavonoids shaped the seasonal composition of gut microbiota as an important driving force in diets. The gut microbiota is exposed to more diverse and abundant flavonoids in the leaf eating stage. Flavonoids are well-known antibacterial PSMs, because they can kill or inhibit bacterial cells in a variety of ways, such as by causing membrane disruption and inhibition of nucleic acid synthesis, as well as by inhibiting bacterial virulence [24, 44, 45]. When the giant panda feeds on high-flavonoid bamboo leaves, its gut microbiota has lower gene and species diversity. That is, many species present in the shoot eating stage are very sensitive to flavonoids, whereas those others in the leaf eating stage have greater flavonoid conversion potential. Previous studies have shown that gut microbes play a key role in the biotransformation of flavonoids; however, the arbitrariness, culture dependency in the selection of bacterial species, and the commercial availability of flavonoids make it difficult to uncover the general correlation between flavonoids and the gut microbial community. Here, the microbiota of wild and captive giant pandas has a differentiated microbial community structure at the phylum level. Integrated analysis showed microbes within *Proteobacteria* may be inhibited by the antibacterial property of flavonoids, whereas more members belonging to *Firmicutes* are favored selectively by the specific nutrients in flavonoids [46].

In addition to the effects of flavonoids on the composition of gut microbiota, we furthermore found the beneficial influence on the functions of microbial community. In the wild, the giant panda feeds on bamboo leaves for 8 months of the year, from September to April [47]. During this period, all the nutrition and energy of giant pandas for basic maintenance metabolism and effective immunological function, as well as regular breeding (mating and birth), come from the leaves of *B. fargesii* [47]. That is, compared with the shoot eating stage, the gut microbiota in the leaf eating stage is of lower diversity, but it remains healthier for the giant panda. It has been reported that captive pandas in the shoot eating season have higher levels of ketone bodies, lactate, urea, blood urea nitrogen, and creatinine, as well as white blood cells and neutrophils, than those in the leaf eating season, indicating a potential inflammatory reaction in the shoot season [48]. As evidenced by the lower abundance of virulence factors in the leaf eating season than in the shoot eating season, we suggest that this inflammation may be induced by gut microbial pathogens. Therefore, bamboo shoots may compensate for primary macronutrients, especially the urgent need for protein after the energy-intensive mating season [49]. However, it also provides more space for opportunistic pathogens. Consistent with a previous report [21], captive pandas encounter more VFs than wild pandas, and the differences in VFs between the two seasons are more distinct for the captive population. Thus, the decreased abundance of VFs with higher flavonoid intakes in leaf eating stage indicated that bamboo leaves can be significant sources of macronutrients, such as essential proteins, but also medicaments for giant pandas, such as flavonoids which play beneficial roles by targeting gut microbial community. As many clinical pathogens pose a threat to the health of the giant panda population [8], diverse flavonoids in bamboo leaves may be candidate antagonistic agents against pathogens.

In conclusion, our results provide an overview of the flavonoid metabolic profile and reveal the interactions between flavonoids and the gut microbiota, which plays substantial roles in host health maintenance by shaping the microbiota configuration, suggesting that dietary flavonoids could serve as prebiotics in the conservation management of wild animals.

## Materials and methods

### Samples

In the wild, all bamboo leaves or shoots were sampled along with fecal collection at the same location. Feces were collected immediately after defecation in triplicate (two for metabolome determination and one for metagenomic analysis). In captive settings, all bamboo diets (leaves or shoots), feces and plasma were sampled at the same time for each individual. Plasma from captive pandas was separated within 2 h by centrifugation. All the diet, feces and plasma samples were snap frozen with liquid nitrogen, shipped to the laboratory on dry ice, and stored at − 80 °C until use.

### Metabolic profiling of targeted flavonoids in food, feces, and plasma

To study the absorption and metabolism of flavonoids in giant pandas, we used widely targeted metabolomics for quantitative analysis of flavonoids in diets, feces, and plasma. Briefly, 100 mg of freeze-dried samples was crushed and extracted with aqueous methanol. Following centrifugation, the extracts were absorbed and filtered. Next, the sample extracts were analyzed using a liquid chromatography-electrospray ionization-tandem mass spectrometry (LC-ESI-MS/MS) system (UPLC, Shim-pack UFLC SHIMADZU CBM30A system, http://www.shimadzu.com.cn/; MS, Applied Biosystems 6500 QTRAP, http://www.appliedbiosystems.com.cn/). LC-based separation and multiple-reaction monitoring (MRM) transitions were optimized using a local library of reference compounds to achieve absolute quantification on a triple quadrupole MS system (QqQ). Quantification of metabolites was carried out using a scheduled MRM method [18]. Details are available in the Supplementary Methods.

### Metabolic profiling of non-flavonoid compounds (NFCs) in feces

To characterize the full metabolic outcomes of the intestinal microenvironment and distinguish flavonoids from non-flavonoid compounds (NFCs), we also detected 112 NFCs (Table S7) in feces by ultra-performance liquid chromatography coupled to tandem mass spectrometry (UPLC-MS/MS; ACQUITY UPLC-Xevo TQ-S, Waters Corp., Milford, MA, USA). Details are available in the Supplementary Methods.

### Metagenomic analysis

DNA was isolated by a bead-beating procedure with the QIAamp PowerFecal DNA Kit (Qiagen, Germany). The pretreatment protocol prior to DNA extraction was as described by Xue et al. (2015) [41]. All samples were sequenced on the Illumina platform (paired-end; insert size, 350 bp; read length, 150 bp). Adaptor and low-quality reads were discarded from the raw reads, and the remaining reads were filtered in order to eliminate giant panda host DNA. High-quality paired-end reads from each sample were used for de novo assembly with IDBA_UD [50] and Megahit [51] into contigs of at least 300 bp. Genes were predicted using MetaGene [52]. A nonredundant gene catalog was constructed with CD-HIT using the parameters (identity ≥ 0.9, coverage ≥ 0.9) [53]. All the genes were clustered into co-abundance gene groups (CAGs) based on their abundance using the canopy-based algorithm with default parameters [22]. CAGs with > 700 genes were regarded as metagenomic species (MGS) for further analyses [22]. More details on rarefaction curve analysis, gene richness, and diversity, and functional annotation are available in the Supplementary Methods.

### Fecal bacteria culture in vitro with flavonoids

Fresh fecal samples (three mixed samples of the same day) from a captive giant panda named Meng Meng were collected within three consecutive days from the Beijing Zoo. Fecal slurry was prepared by diluting the fecal sample in sterile PBS medium, stored in an anaerobic bag and shipped to the laboratory. One hundred microliters of bacterial suspension was inoculated onto thiolglycollate medium (Qingdao Hope Bio-Technology Co., Ltd., China) agar plates for the treatment group (flavonoid concentration, 2% w/w) and control group. Each group had three replications. After 24 h of culture at 37 °C under anaerobic conditions, samples were collected for genomic DNA extraction. The V3-V4 region of the bacterial 16S rRNA gene was amplified for analysis.

### Statistical analysis

The identification of utilization preference of flavonoids by giant pandas is based on the relative changes in percentages between diets and matched feces. This method is independent of absolute digestive efficiencies of separate compounds [47, 54]. The differential preferences may be made out along with following three gradually progressive levels: firstly, plasma metabolome showed that dietary flavonoids were determined to be absorbed by the giant panda; secondly, if all flavonoids could get coordinated proportion of digestive extraction efficiencies in giant pandas, the percentages of all subclasses/monomers in feces would stay unchanged with diets; finally, if they are utilized with varying degrees, the percentages of specific subclasses/monomers in feces would appear to be fluctuated.

We used *t*-tests to compare the ratios of flavonoids in the foods to matched fecal samples to establish relative utilization preference (as explained above). Levene’s test was used to test for equality of variances, and where the null hypothesis of equal variances was rejected, we applied Wilcoxon rank sum tests that does not assume equal variances.

Seasonal differences in metabolites were identified using a multi-criterion assessment based on the Wilcoxon rank-sum test (*P* < 0.01, FDR < 0.05) and strength of contribution (variable importance in projection, VIP > 1) in an orthogonal partial least square discriminant analysis (OPLS-DA) model. Significant differences in the metagenome between seasons were tested using the Wilcoxon rank-sum test (*P* < 0.01, FDR < 0.05).

To investigate whether the altered abundance of metabolites correlated with the altered gut microbiota, we used co-inertia analysis (CoIA) to find covariation between the fecal metabolome and metagenome. CoIA is a multivariate statistical method that can be used for data integration between heterogeneous datasets and aims to identify trends or relationships between parallel datasets [55, 56]. Here, it was performed on fecal metabolites and metagenomic gene clusters using the R packages ade4 and omicade4, and the Spearman rank correlation coefficient was used to assess the correlation between the metabolome and metagenome corrected for multiple testing using the R package psych.

Linear discriminant analysis effect size (LEfSe) analyses were used to identify different bacterial features in vitro between the treatment (with additive flavonoids) and control groups (LDA score > 2).

Unless otherwise stated, all statistical analyses were performed in and all the plots were generated by R software (3.5.1). The associative network between flavonoids and MGS (Fig. 3G) was visualized with Cytoscape 3.7.0 software.

## Supplementary Information

**Additional file 1: Supplemental Materials and Methods.** Supplemental References. **Figure S1.** Gut microbial alterations with diet-associated seasons. **Figure S2.** The composition of microbiota at the level of phylum and genus. **Figure S3.** The covariation of gene richness and MGS number per sample. **Figure S4.** The seasonal differences of metagenomic species. **Figure S5.** Seasonally different metabolites in fecal samples. **Figure S6.** Heatmap of different metabolites in feces between seasons. **Figure S7.** Associations of fecal metabolome with metagenome for captive pandas. **Figure S8.** Associations of fecal metabolome with metagenome for wild pandas. **Figure S9.** Seasonal differences of genes involved quorum sensing. **Figure S10.** The seasonal differences of microbial virulence factors (VFs). **Table S1.** Dietary bamboos and habitual grouping information. **Table S2.** All flavonoids detected in all sample types (dietary bamboos, feces and plasma). **Table S3.** Qualitative determination of all flavonoids. **Table S4.** Monomers with different proportion in diet and feces. **Table S5.** Sample information for metagenomic analysis and assembly information. **Table S6.** All the 144 MGS. **Table S7.** All the 112 nonflavonoid compounds (NFCs) in feces.
