# Supplementary material for: Multi-omics reveals the positive leverage of plant secondary metabolites on the gut microbiota in a non-model mammal
Source: Microbiome. 2021 Sep 22;9:192. doi: 10.1186/s40168-021-01142-6 (PMC8456708; doi:10.1186/s40168-021-01142-6)
Supplement: Supplementary file 2 — Additional file 1: Supplemental Materials and Methods. Supplemental References. Figure S1. Gut microbial alterations with diet-associated seasons. Figure S2. The composition of microbiota at the level of phylum and genus. Figure S3. The covariation of gene richness and MGS number per sample. Figure S4. The seasonal differences of metagenomic species. Figure S5. Seasonally different metabolites in fecal samples. Figure S6. Heatmap of different metabolites in feces between seasons. Figure S7. Associations of fecal metabolome with metagenome for captive pandas. Figure S8. Associations of fecal metabolome with metagenome for wild pandas. Figure S9. Seasonal differences of genes involved quorum sensing. Figure S10. The seasonal differences of microbial virulence factors (VFs). Table S1. Dietary bamboos and habitual grouping information. Table S2. All flavonoids detected in all sample types (dietary bamboos, feces and plasma). Table S3. Qualitative determination of all flavonoids. Table S4. Monomers with different proportion in diet and feces. Table S5. Sample information for metagenomic analysis and assembly information. Table S6. All the 144 MGS. Table S7. All the 112 nonflavonoid compounds (NFCs) in feces. [file 40168_2021_1142_MOESM2_ESM.pdf]

Supplementary Materials

Multi-omics reveals the positive leverage of plant secondary metabolites on the gut microbiota in a non-model mammal

Le Wang[1,2†], Guangping Huang[1†], Rong Hou[3†], Dunwu Qi[3], Qi Wu[1], Yonggang Nie[1,2], Zhenqiang Zuo[5], Rui Ma[3], Wenliang Zhou[1,4], Yingjie Ma[1,2], Yibo Hu[1,2], Zhisong Yang[6], Li Yan[1], Fuwen Wei[1,2,4*]

*Corresponding author. Email: weifw@ioz.ac.cn

This PDF file includes:

Supplemental Materials and Methods

Supplemental References

Fig. S1. Gut microbial alterations with diet-associated seasons.

Fig. S2. The composition of microbiota at the level of phylum and genus.

Fig. S3. The covariation of gene richness and MGS number per sample.

Fig. S4. The seasonal differences of metagenomic species.

Fig. S5. Seasonally different metabolites in fecal samples.

Fig. S6. Heatmap of different metabolites in feces between seasons.

Fig. S7. Associations of fecal metabolome with metagenome for captive pandas.

Fig. S8. Associations of fecal metabolome with metagenome for wild pandas.

Fig. S9. Seasonal differences of genes involved quorum sensing.

Fig. S10. The seasonal differences of microbial virulence factors (VFs).

Table S1 Dietary bamboos and habitual grouping information.

Table S2 All flavonoids detected in all sample types (dietary bamboos, feces and plasma).

Table S3 Qualitative determination of all flavonoids.

Table S4 Monomers with different proportion in diet and feces.

Table S5 Sample information for metagenomic analysis and assembly information.

Table S6 All the 144 MGS.

Table S7 All the 112 nonflavonoid compounds (NFCs) in feces.

Supplementary Materials and Methods

Study site, subjects and sample collection

Sampling in the wild. All the wild dietary bamboos and fecal samples were collected in Foping National Natural Reserve, Shaanxi, Qinling Mountains. Samples were collected from November of 2017 to May of 2018. Following their diet, samples were divided into two groups: WL (collected in winter (from November to next March) when feeding on the leaves of *Bashania fargesii*) and WS (collected in April and May when feeding on shoots of *B. fargesii*). All fecal samples were collected by direct tracking. We adopted the strictest criteria for fresh samples: either our skilled seekers have seen the giant panda, or the feces was of residual heat. With the surfaces in contact with environment excluded, feces were collected immediately after defecation snap-frozen in liquid nitrogen and shipped to the laboratory on dry ice. Diet samples were collected at every site where fresh feces were sampled.

Sampling in captivity. All the captive dietary bamboos, feces and plasma samples were collected with approval of Institutional Animal Care and Use Committee in Chengdu Research Base of Giant Panda Breeding, Sichuan, China. Samples were collected from May of 2018 to February of 2019, in which May and June of 2018 correspond to period of bamboo shoot-eating stage (CS_I: collected in May when feeding on *Pleidolastus amarus*; CS_II: collected in June when feeding on *Phyllostachys nidularia*), December and January correspond to period of bamboo leaves-eating (CL: when feeding on *B. fargesii*). Totally, 27 adult giant pandas in good health (15 females and 12 males) were enrolled, of which no subjects were fed antibiotics within the previous month. 23

pandas were captive-born and 4 were wild-born but captive-fed 8-14 years.

Metabolomics profiling of food, feces and plasma samples

Targeted flavonoids profiling

Sample extract. The freeze-dried samples were crushed using a mixer mill (MM400, Retsch, Laichi, Germany) with a zirconia bead for 1.5 min at 30 Hz. 100 mg of powder was weighed and extracted overnight at 4°C with 1.0 mL 70% aqueous methanol. Following centrifugation at 10,000 × g for 10 min, the extracts were absorbed (CNWBOND Carbon-GCB SPE Cartridge, 250 mg, 3 mL; ANPEL, Shanghai, China) and filtrated (SCAA-104, 0.22 μ m pore size; ANPEL, Shanghai, China) before LC-MS analysis.

Mass spectrometry (MS). The sample extracts were analyzed using an LC-ESI-MS/MS system (UPLC, Shim-pack UFLC SHIMADZU CBM30A system; MS, Applied Biosystems 6500 QTRAP). LIT and triple quadrupole (QQQ) scans were acquired on a triple quadrupole-linear ion trap mass spectrometer (Q TRAP), API 6500 Q TRAP LC/MS/MS System, equipped with an ESI Turbo Ion-Spray interface, operating in a positive ion mode and controlled by Analyst 1.6.3 software (AB Sciex, Waltham, MA, USA). Liquid chromatography (LC)-based separation and multiple-reaction-monitoring (MRM) transitions were optimized using a local library of reference compounds to achieve absolute quantification on triple quadrupoles MS (QqQ). The presence of flavonoids was determined using a UPLC-ESI-MS/MS-based, widely targeted metabolome. Based on the self-built database MWDB (Metware Biotechnology Co., Ltd. Wuhan, China) and public database of metabolite information,

the metabolites of the samples were qualitatively and quantitatively analyzed by MS. The characteristic ions of each substance were screened out by the triple quadrupole rod, and the signal strength of the characteristic ions were obtained in the detector. The MS file under the sample was opened with MultiaQuant software 3.0.3 to carry out the integration and correction of chromatographic peaks, and the relative content of the corresponding substances in the peak area of each chromatographic peak were calculated. Finally, all chromatographic peak area integral data were derived. In order to compare the contents of each metabolite in different samples, we calibrated the mass spectrum peaks detected by each metabolite in different samples based on the information of metabolite retention time and peak pattern(*1*).

Metabolic profiling of non-flavonoids compounds (NFCs). Fecal samples were thawed on ice-bath to diminish degradation. About 10mg of each sample was weighted and transferred to a new 1.5mL tube. 25μL of water was added and the sample was homogenated with zirconium oxide beads for 3 minutes. 185μL of ACN/Methanol (8/2) was added to extract the metabolites. The sample was centrifuged at 18000g for 20 minutes. Then the supernatant was transferred to a 96-well plate. The following procedures were performed on a Biomek 4000 workstation (Biomek 4000, Beckman Coulter, Inc., Brea, California, USA). 20μL of freshly prepared derivative reagents was added to each well. The plate was sealed and the derivatization was carried out at 30°C for 60 min. After derivatization, 350μL of ice-cold 50% methanol solution was added to dilute the sample. Then the plate was stored at -20°C for 20 minutes and followed by 4000g centrifugation at 4 °C for 30 minutes. 135μL of supernatant was transferred to a

new 96-well plate with 15μL internal standards in each well. Serial dilutions of derivatized stock standards were added to the left wells. Finally, an ultra-performance liquid chromatography coupled to tandem mass spectrometry (UPLC-MS/MS) system (ACQUITY UPLC-Xevo TQ-S, Waters Corp., Milford, MA, USA) was used to quantitate the microbial metabolite.

Metagenomic analysis

Rarefaction curve analysis, gene richness and diversity. To assess gene richness in all samples, we generated a rarefaction curve. For a given number of samples, we performed a randomized sampling n times (n is the sample number) in each group, respectively, and estimated the total number of genes that could be identified from these samples.

Functional annotation, KO profiles and virulence factor profiles. To annotate the metagenomes, we aligned the nonredundant gut microbiome gene set against the Kyoto Encyclopedia of Genes and Genomes (KEGG) database using BLASTP with 1e-5 and "single-directional best hit". The identification of virulence genes was performed by comparing the annotated protein sequences of the non-redundant gene set with BLASTP against the VFDB database (full dataset). Hits with an e-value < 1e-5, percent identity > 80%, and alignment length >50 bp were considered positive.

Association between metagenome and metabolome

Co-inertia analysis (CoIA) was performed on MGS abundance profiles and metabolite abundance profiles of two seasonal samples to assess the relationship between MGS and metabolites that differed in abundance between seasons. The

associations of metabolites and MGS were established by R software (3.5.1, psych package), and the plots were generated by R software (3.5.1, vegan package).

Fecal culture *in vitro* with bamboo flavonoids

Fresh feces were collected immediately after defecation from Beijing Zoo. The subject giant panda was an adult female named Meng Meng (Stud# 822). It mainly fed on the bamboo leaves of *Phyllostachys propinqua McClure*. Fecal bacterial suspension were prepared in triplicate for following culture. The culture medium was supplied by Qingdao Hope Bio-Technology Co., Ltd (Qingdao, China). Its formula is as follows (g/L): Peptone from Casein, 15.0; Yeast Extract, 5.0; D(+)glucose, 5.5; Sodim Thioglycollate, 0.5; L-Cystine, 0.5; Sodium Chloride, 2.5; Sodium Resazurin, 0.001; Agar, 0.75; Final pH7.1±0.2 at 25°C. Bamboo flavonoids were produced by Bioruler (No: RH42691). 100 μl of bacterial suspension were inoculated, following by 24-h culture at 37 °C under anaerobic conditions.

Bacteria were rinsed by 1ml of sterile PBS following by centrifugation. Genomic DNA was extracted QIAamp PowerFecal DNA Kit (Qiagen, Germany). The v3-v4 region of the bacterial 16S rRNA gene was amplified with the following primers (27F: 5'- ACTCCTACGGGAGGCAGCAG-3' and 806R: 5'-GGACTACHVGGGTWTCTA AT-3'). Sequencing of the pooled amplicons was conducted by Majorbio Bio-Pharm Technology Co. Ltd. (Shanghai, China). Barcodes and sequencing primers were trimmed before assembly. The data were analyzed on the free online platform of Majorbio Cloud Platform (www.majorbio.com).

Supplemental References

1. Chen W, Gong L, Guo ZL, Wang WS, Zhang H, Liu XQ, et al. A novel integrated method for large-scale detection, identification, and quantification of widely targeted metabolites: application in the study of rice metabolomics. Mol Plant. 2013; 6: 1769–80.

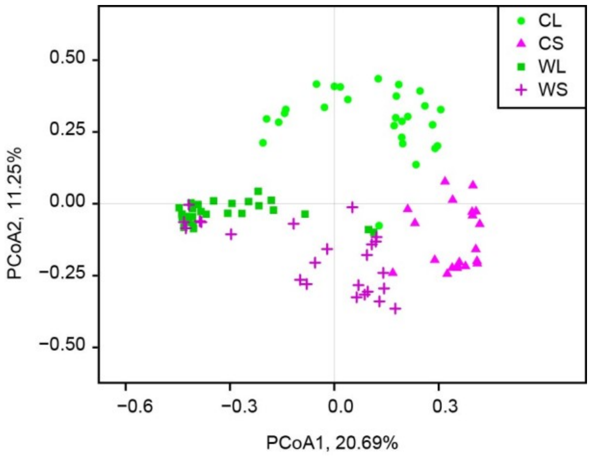

Fig. S1. Gut microbial alterations with diet-associated seasons. Seasonal differential genes separated the seasonal groups for both the wild and captive pandas. Principal coordinates analysis was performed on the basis of the Bray-Curtis distance for seasonally different genes ($P < 0.01$, Wilcoxon rank sum test, FDR < 0.05). PCoA1, principal coordinate 1; PCoA2, principal coordinate 2.

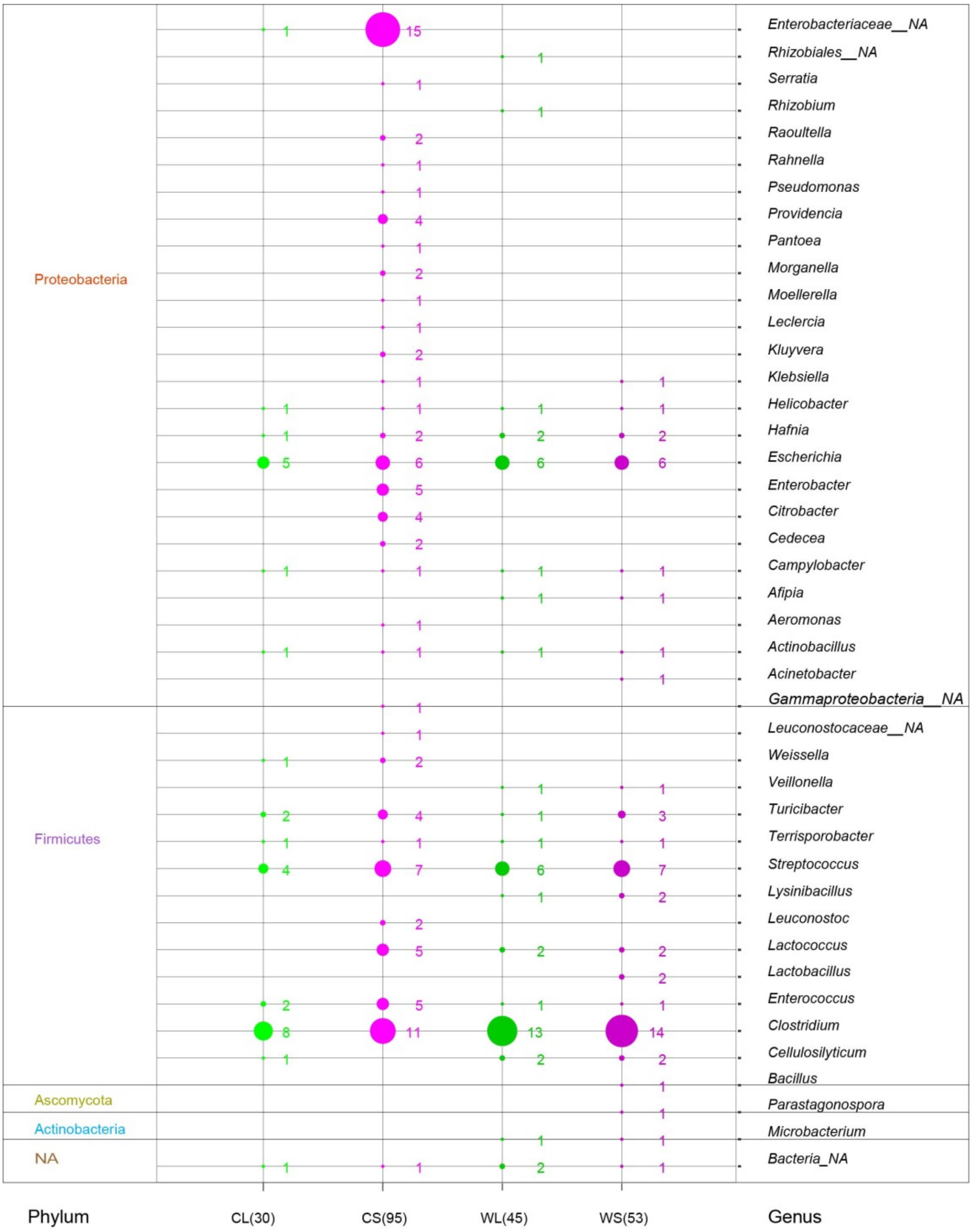

Fig. S2. The composition of microbiota at the level of phylum and genus. The size of bubbles indicated the number of MGS (figures on the right) in the corresponding genus. MGS which were not identified were labeled with "NA". No bubble at one node indicated no MGS belonging to the genus were detected in the given group. The figures along with the group names referred to the total number of MGS in each group.

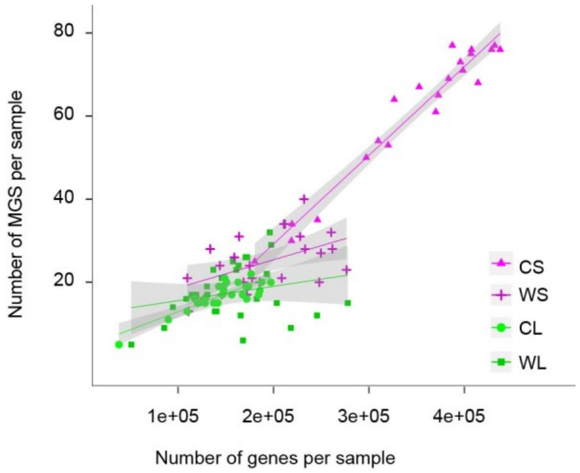

Fig. S3. The covariation of gene richness and MGS number per sample. MGS, Metagenomic species. A linear fitting (the solid lines in four colors and the 95% confidence interval in gray) of the numbers of MGS versus gene numbers showed the positive correlations between gene and MGS richness (WL: P = 0.2061, WS: P = 0.0115*, CL: P = 4.01e-8***, CS: P = 8.45e-14***, * 0.01 < P value < 0.05, ***P value< 0.001).

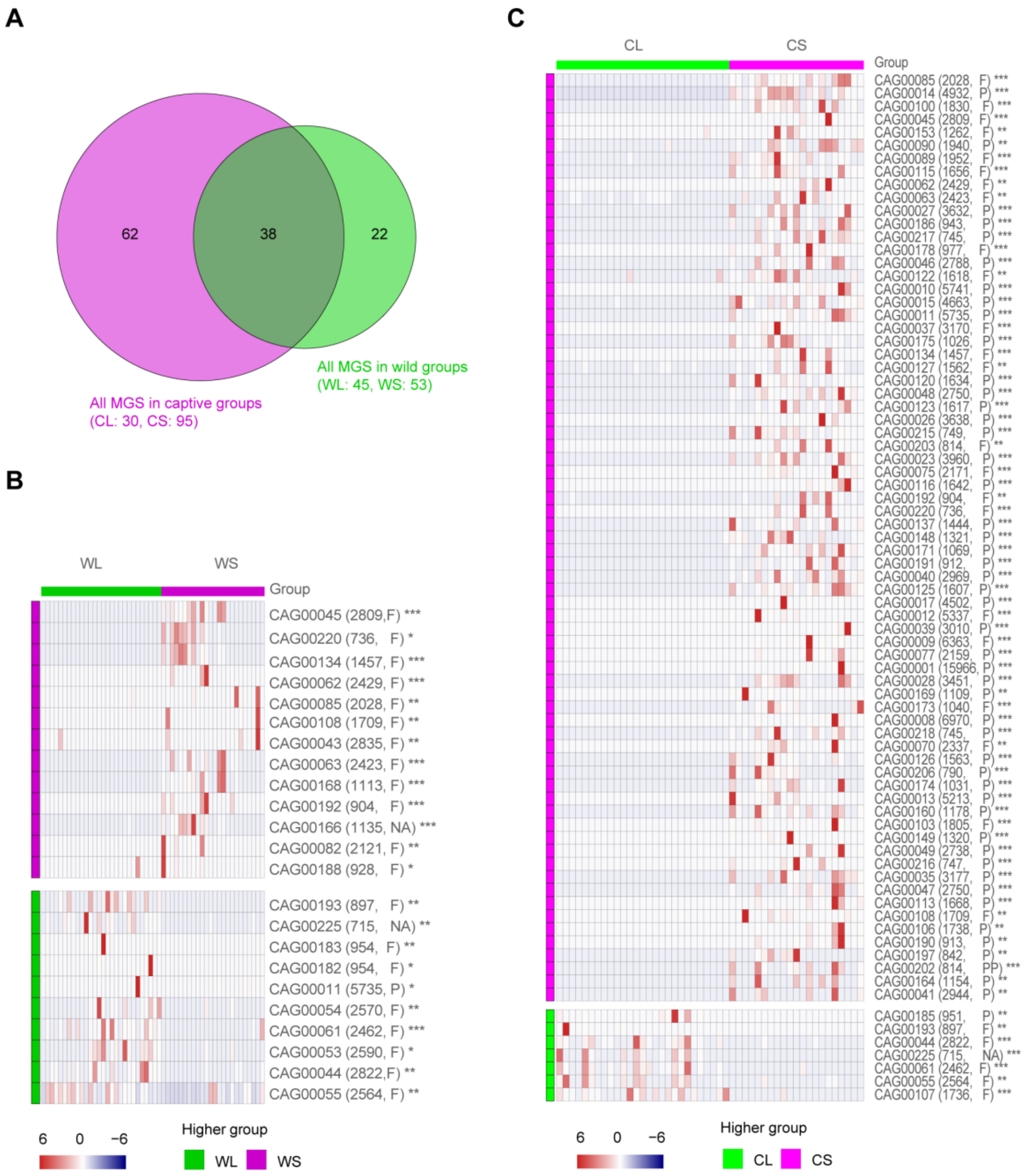

Fig. S4. The seasonal differences of metagenomic species. (A) Venn plot of metagenomic species (MGS) of wild and captive groups. **(B-C)** Heatmap of differential MGS between leaf season and shoot season in wild **(B)** and captivity **(C)**. For **B** and **C**, the numbers in parentheses next to each MGS number represent the gene number of this MGS, and the following capital letter indicate the corresponding phylum: F, Firmicutes; P, Proteobacteria; NA, not identified. * 0.01 < *P* value < 0.05, ****P* value< 0.001.

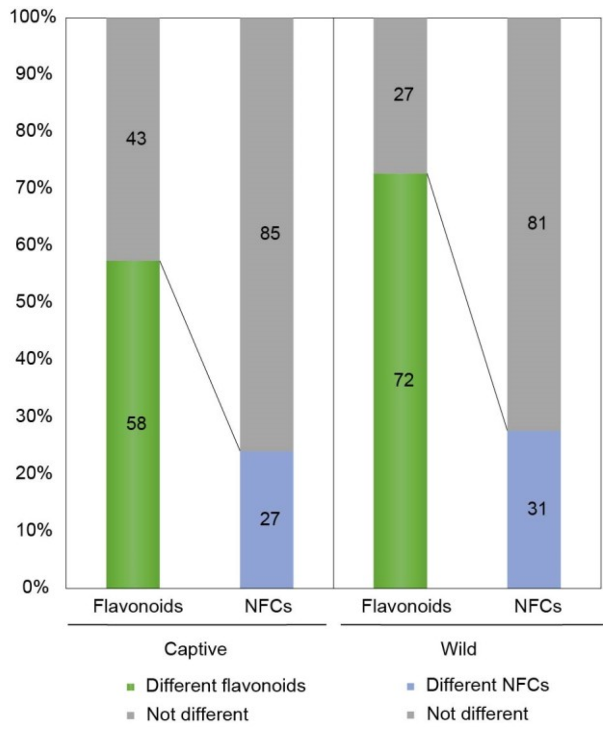

Fig. S5. Seasonally different metabolites in fecal samples. The composition of seasonally different flavonoids and cometabolites ($P < 0.01$, Wilcoxon rank sum test, FDR < 0.05, VIP >1) with regard to the proportion of total number. The figures of different metabolites were labeled in the bar.

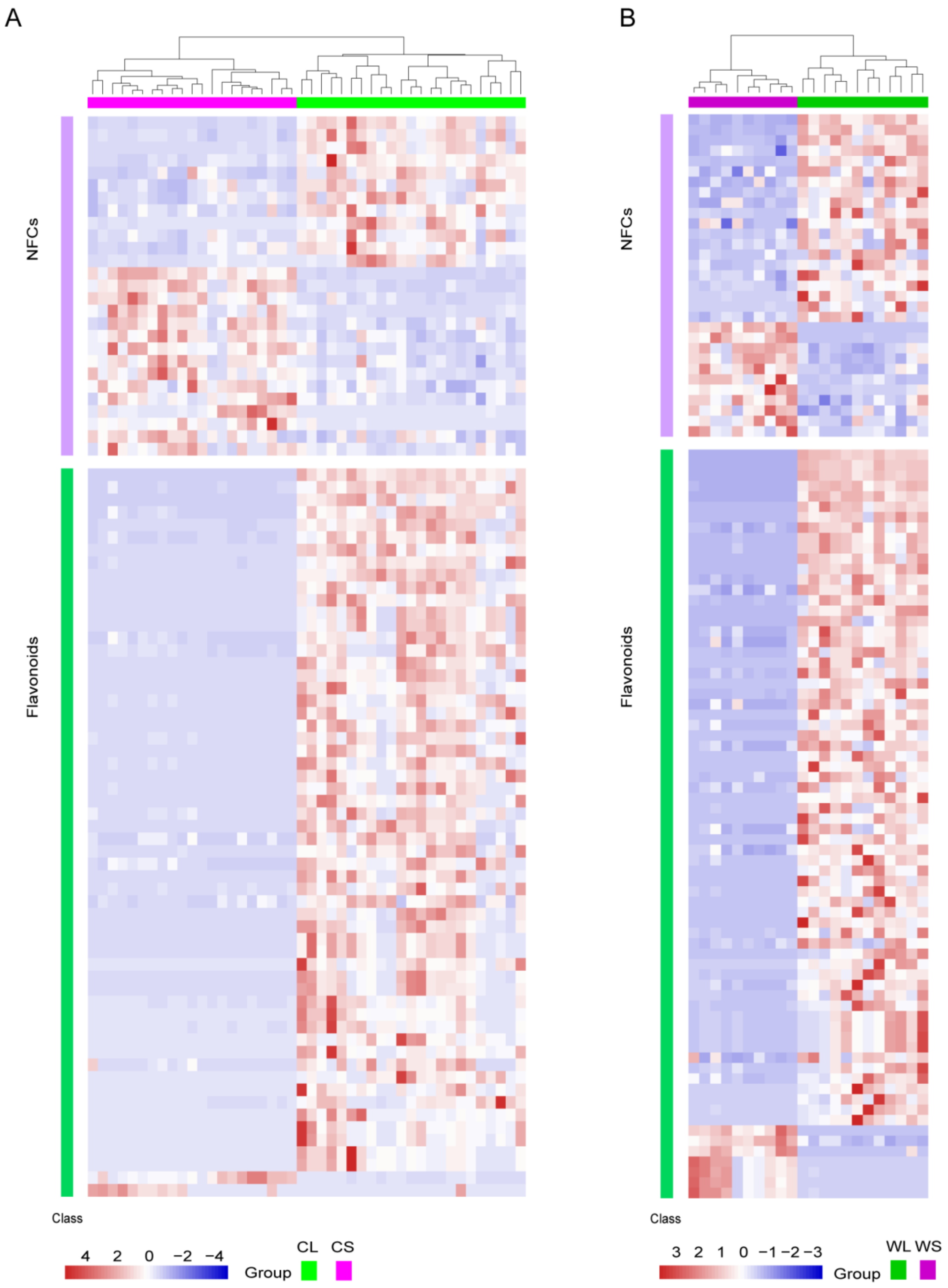

Fig. S6. Heatmap of different metabolites in feces between seasons. (A) Captive groups. **(B)** Wild groups.

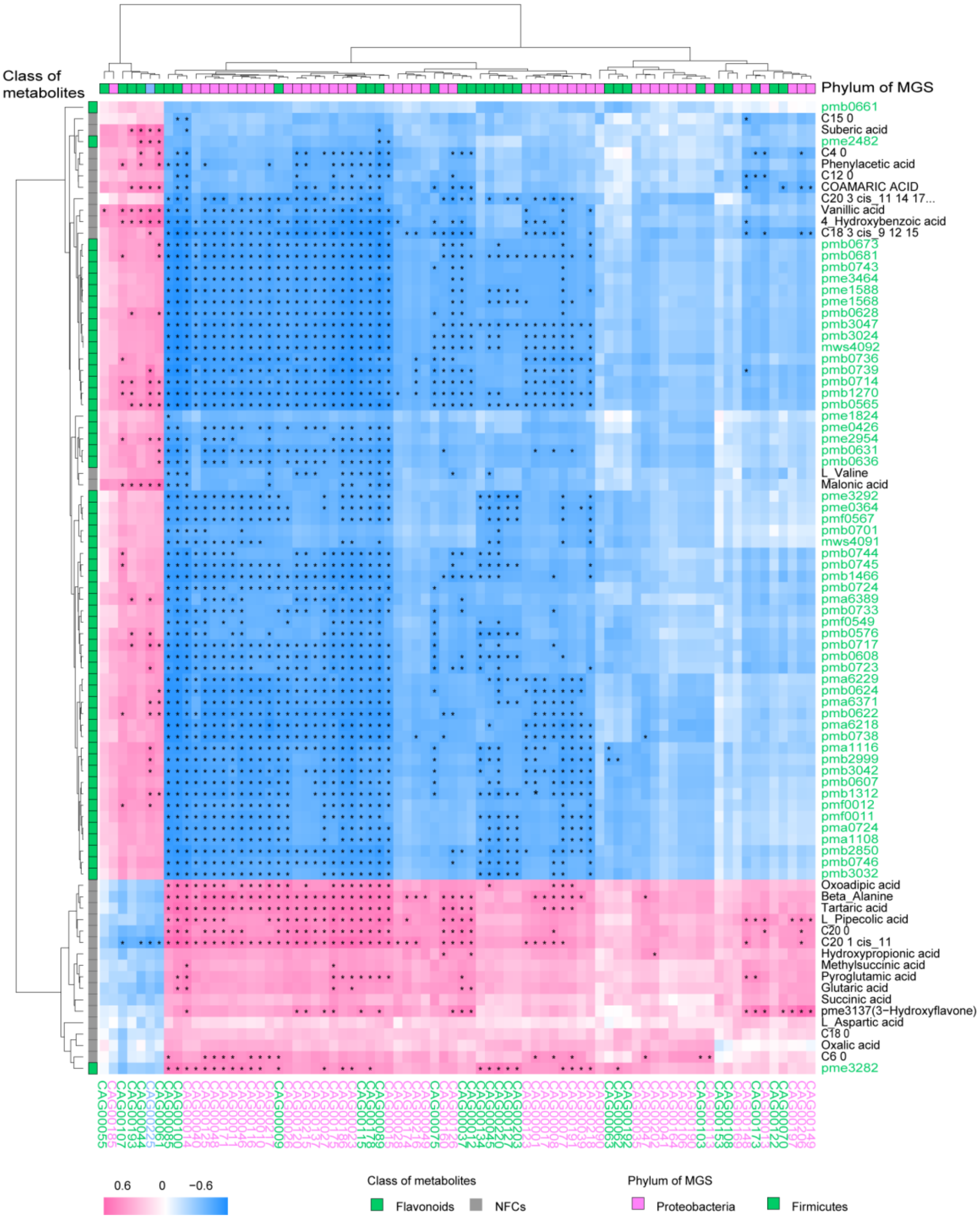

Fig. S7. Associations of fecal metabolome with metagenome for captive pandas. The heat map of the Spearman's rank correlation coefficient between fecal metabolites (P < 0.01, Wilcoxon rank sum test, FDR < 0.05, VIP >1) and metagenomic species (MGS) (P < 0.01, Wilcoxon rank sum test, FDR < 0.05). *P < 0.01, |r| > 0.6; Spearman's rank correlation. Metabolites in green and black denote flavonoids (n=58)

and NFCs (n=27), respectively. MGS in green and pink denote Firmicutes (n=28) and Proteobacteria (n=49).

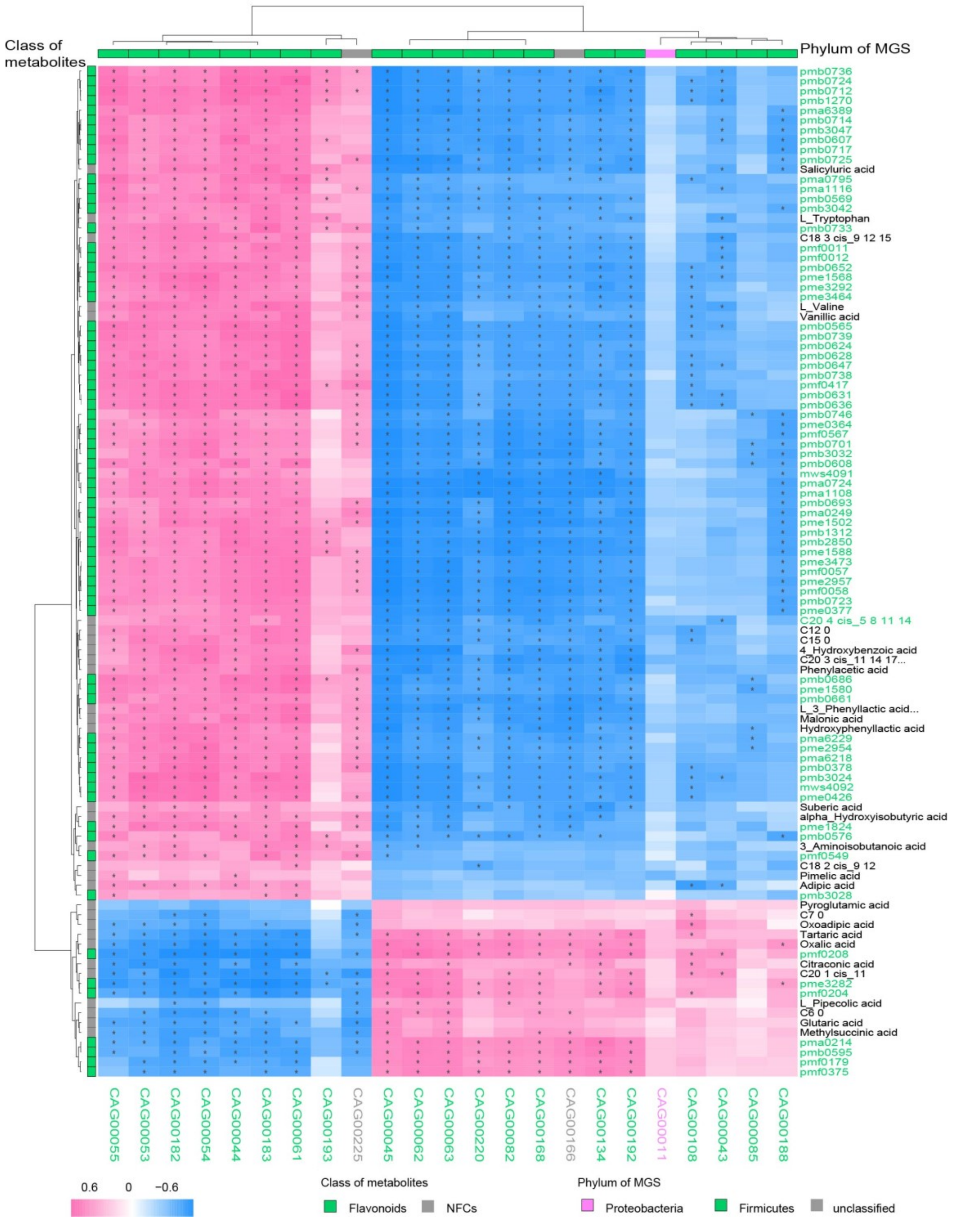

Fig. S8. Associations of fecal metabolome with metagenome for wild pandas. The heat map of the Spearman's rank correlation coefficient between fecal metabolites ($P < 0.01$, Wilcoxon rank sum test, FDR < 0.05, VIP >1) and metagenomic species (MGS) ($P < 0.01$, Wilcoxon rank sum test, FDR < 0.05). *$P < 0.01$, $|r| > 0.6$; Spearman's rank correlation. Metabolites in green and black denote flavonoids (n=72) and NFCs (n=31),

respectively. MGS in green and pink denote Firmicutes (n=20) and Proteobacteria (n=1).

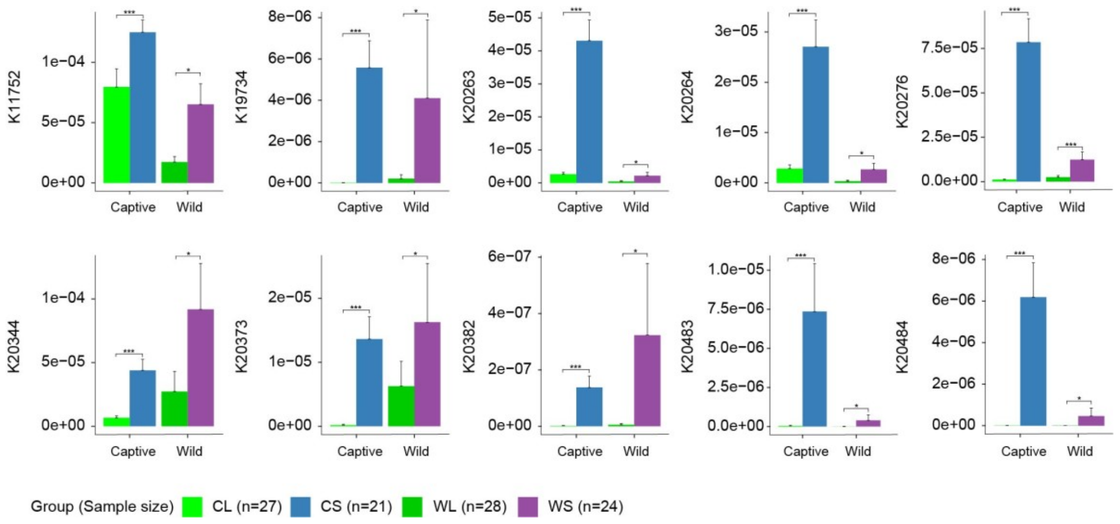

Fig. S9. Seasonal differences of genes involved quorum sensing. Only 10 KOs shared by wild and captive samples were shown. Two-tailed Wilcoxon rank-sum test was used to determine significance. * 0.01 < FDR < 0.05, *** FDR < 0.001.

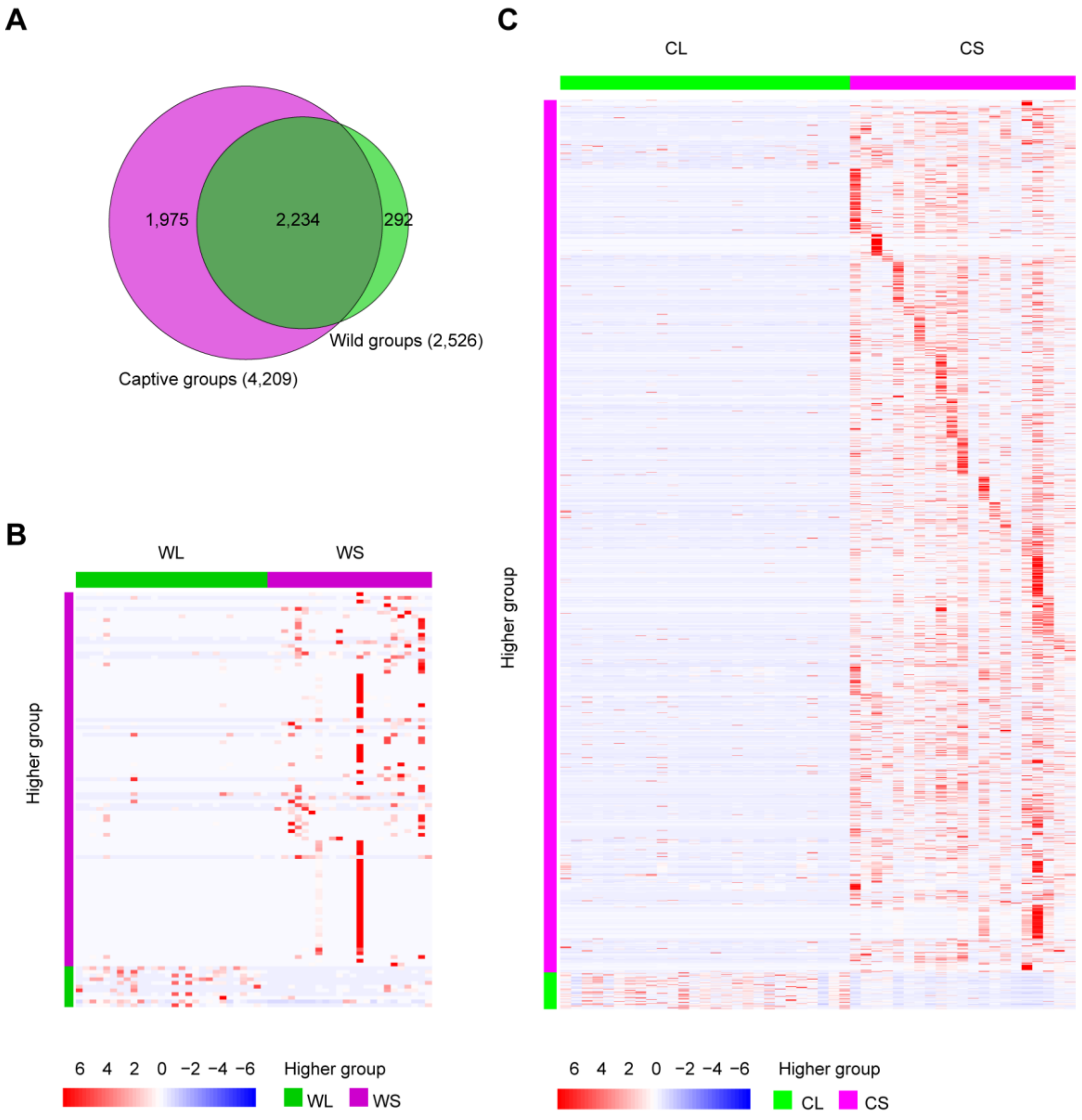

Fig. S10. The seasonal differences of microbial virulence factors (VFs). (A) Venn plot of genes annotated as VFs. **(B-C)** Heatmap of differential genes involving VFs between leaf season and shoot season in wild groups **(B)** and captive groups **(C)**.

Table S1 Dietary bamboos and habitual grouping information.

Group	Subgroup	Bamboo species	Bamboo part selected by giant pandas	Source	Sample size (n)
CL	CL	*Bashania fargesii*	Leaf	Captive (Chengdu base)	3 *
CS	CS_I	*Pleidolastus amarus*	Shoot	Captive (Chengdu base)	3 *
	CS_II	*Phyllostachys nidularia*	Shoot	Captive (Chengdu base)	3 *
WL	WL	*Bashania fargesii*	Leaf	Wild (Foping)	3 *
WS	WS	*Bashania fargesii*	Shoot	Wild (Foping)	3 *

Chengdu base: Chengdu Research Base of Giant Panda Breeding. Foping: Foping National Natural Reserve. *For both captive and wild dietary bamboos, 3 mixed samples went through extraction process for flavonoids determination. In captivity, one diet sample (about 5g fresh weight) was collected for each giant panda. For wild food bamboos, samples were collected from multiple sites where fresh droppings were collected.

Table S2 All flavonoids detected in all sample types (dietary bamboos, feces and plasma).

Index	Q1 (Da)	Q3 (Da) MS only	Rt (min)	Molecular Weight (Da)	Ionization model	Compounds	Class
mws4091	4.31E+02	3.11E+02	3.90E+00	4.32E+02	[M-H]-	Vitexin(Apigenin 8-C-glucoside)	Flavone
mws4092	4.47E+02	3.27E+02	3.58E+00	4.48E+02	[M-H]-	Orientin(Luteolin-8-C-glucoside)	Flavone
pma0214	4.79E+02	3.17E+02	3.91E+00	4.78E+02	[M+H]+	methylQuercetin O-hexoside	Flavonol
pma0249	4.79E+02	3.17E+02	3.52E+00	4.78E+02	[M+H]+	Selgin 5-O-hexoside	Flavone
pma0724	4.35E+02	2.85E+02	3.79E+00	4.34E+02	[M+H]+	Naringenin C-hexoside	Flavanone
pma0787	5.51E+02	1.81E+02	4.63E+00	5.50E+02	[M+H]+	Quercetin-3-(6"-malonyl)-Glucoside	Flavonol
pma0791	5.21E+02	2.73E+02	4.50E+00	5.20E+02	[M+H]+	Naringenin O-malonylhexoside	Flavanone
pma0795	5.51E+02	3.03E+02	4.56E+00	5.50E+02	[M+H]+	Tricetin O-malonylhexoside	Flavone
pma1108	4.33E+02	2.83E+02	3.78E+00	4.32E+02	[M+H]+	Apigenin C-glucoside	Flavone

pma1116	3.01E+02	2.86E+02	4.13E+00	3.00E+02	[M+H]+	Kaempferide	Flavonol
pma6218	4.19E+02	3.83E+02	3.84E+00	4.18E+02	[M+H]+	O-methylnaringenin C-pentoside	Flavanone
pma6229	4.51E+02	3.31E+02	3.37E+00	4.50E+02	[M+H]+	Eriodictyol C-hexoside	Flavanone
pma6371	6.11E+02	4.73E+02	3.08E+00	6.10E+02	[M+H]+	"di-C,C-hexosyl-luteolin"	Flavone
pma6389	3.45E+02	1.77E+02	6.33E+00	3.44E+02	[M+H]+	Ayanin	Flavonol
pma6520	8.36E+02	3.31E+02	4.21E+00	8.35E+02	[M+H]+	Tricin 4'-O-(β-guaiacylglyceryl) ether-O-rutinoside	Flavone
pma6638	4.77E+02	3.15E+02	5.30E+00	4.76E+02	[M+H]+	O-methylChrysoeriol 7-O-hexoside	Flavone
pmb0378	6.25E+02	1.77E+02	4.47E+00	6.24E+02	[M+H]+	Luteolin O-feruloylhexoside	Flavone
pmb0565	5.09E+02	3.47E+02	3.41E+00	5.08E+02	[M+H]+	Syringetin 3-O-hexoside	Flavonol
pmb0569	5.09E+02	3.47E+02	4.17E+00	5.08E+02	[M+H]+	Syringetin 5-O-hexoside	Flavonol

pmb0576	5.19E+02	2.71E+02	4.23E+00	5.18E+02	[M+H]+	Apigenin O-malonylhexoside	Flavone
pmb0595	4.79E+02	3.17E+02	4.17E+00	4.78E+02	[M+H]+	Isorhamnetin 5-O-hexoside	Flavonol
pmb0607	4.63E+02	3.01E+02	4.30E+00	4.62E+02	[M+H]+	Chrysoeriol 7-O-hexoside	Flavone
pmb0608	5.49E+02	3.01E+02	4.55E+00	5.48E+02	[M+H]+	Chrysoeriol O-malonylhexoside	Flavone
pmb0622	6.11E+02	4.73E+02	3.09E+00	6.10E+02	[M+H]+	C-hexosyl-luteolin O-hexoside	Flavone
pmb0624	6.11E+02	4.31E+02	3.09E+00	6.10E+02	[M+H]+	6-C-hexosyl-luteolin O-hexoside	Flavone
pmb0628	6.13E+02	4.51E+02	3.09E+00	6.12E+02	[M+H]+	Eriodictiol C-hexosyl-O-hexoside	Flavanone
pmb0631	5.81E+02	5.15E+02	3.17E+00	5.80E+02	[M+H]+	C-hexosyl-luteolin C-pentoside	Flavone
pmb0636	5.81E+02	4.31E+02	3.18E+00	5.80E+02	[M+H]+	6-C-hexosyl luteolin O-pentoside	Flavone
pmb0647	5.81E+02	4.19E+02	3.46E+00	5.80E+02	[M+H]+	8-C-hexosyl-luteolin O-pentoside	Flavone
pmb0652	5.65E+02	4.33E+02	3.60E+00	5.64E+02	[M+H]+	C-hexosyl-apigenin O-pentoside	Flavone
pmb0653	5.95E+02	4.63E+02	3.68E+00	5.94E+02	[M+H]+	"di-C,C-hexosyl-apigenin"	Flavone

pmb0661	6.09E+02	4.63E+02	3.87E+00	6.08E+02	[M+H]+	Chrysoeriol C-hexosyl-O-rhamnoside	Flavone
pmb0673	4.03E+02	3.67E+02	4.30E+00	4.02E+02	[M+H]+	Apigenin 6-C-pentoside	Flavone
pmb0681	4.03E+02	3.67E+02	4.32E+00	4.02E+02	[M+H]+	Apigenin 8-C-pentoside	Flavone
pmb0686	5.37E+02	2.89E+02	4.41E+00	5.36E+02	[M+H]+	Eriodictyol O-malonylhexoside	Flavanone
pmb0689	4.63E+02	3.31E+02	4.75E+00	4.62E+02	[M+H]+	Chrysoeriol C-hexoside	Flavone
pmb0693	5.65E+02	4.33E+02	3.39E+00	5.64E+02	[M+H]+	C-hexosyl-apigenin C-pentoside	Flavone
pmb0701	4.63E+02	3.13E+02	3.89E+00	4.62E+02	[M+H]+	Chrysoeriol 8-C-hexoside	Flavone
pmb0712	6.55E+02	3.31E+02	3.50E+00	6.54E+02	[M+H]+	Tricin 5-O-hexosyl-O-hexoside	Flavone
pmb0713	6.55E+02	3.31E+02	3.37E+00	6.54E+02	[M+H]+	Tricin 7-O-hexosyl-O-hexoside	Flavone
pmb0714	6.89E+02	3.31E+02	4.01E+00	6.88E+02	[M+H]+	Tricin 4'-O-(β-guaiacylglyceryl) ether 5-O-hexoside	Flavone

pmb0717	5.27E+02	3.31E+02	4.11E+00	5.26E+02	[M+H]+	Tricin 5-O-β-guaiacylglycerol	Flavone
pmb0723	6.89E+02	3.31E+02	4.97E+00	6.88E+02	[M+H]+	Tricin 4'-O-(β-guaiacylglyceryl) ether O-hexoside	Flavone
pmb0724	4.77E+02	3.31E+02	4.99E+00	4.76E+02	[M+H]+	Tricin O-rhamnoside	Flavone
pmb0725	6.69E+02	3.31E+02	5.10E+00	6.68E+02	[M+H]+	Tricin 7-O-feruloylhexoside	Flavone
pmb0733	6.39E+02	3.31E+02	4.07E+00	6.38E+02	[M+H]+	Tricin 5-O-rutinoside	Flavone
pmb0735	5.07E+02	3.31E+02	4.18E+00	5.06E+02	[M+H]+	Tricin O-glucuronic acid	Flavone
pmb0736	4.93E+02	3.31E+02	4.30E+00	4.92E+02	[M+H]+	Tricin 7-O-hexoside	Flavone
pmb0738	6.99E+02	3.31E+02	4.88E+00	6.98E+02	[M+H]+	Tricin O-sinapoylhexoside	Flavone
pmb0739	6.59E+02	3.31E+02	4.92E+00	6.58E+02	[M+H]+	Tricin O-hexosyl-O-syringin alcohol	Flavone
pmb0743	5.27E+02	3.48E+02	5.44E+00	5.26E+02	[M+H]+	Tricin 7-O-β-guaiacylglycerol	Flavone

pmb0744	4.35E+02	3.31E+02	5.48E+00	4.34E+02	[M+H]+	Tricin O-phenylformic acid	Flavone
pmb0745	4.97E+02	3.31E+02	5.75E+00	4.96E+02	[M+H]+	Tricin 4'-O-syringyl alcohol	Flavone
pmb0746	5.27E+02	3.31E+02	5.84E+00	5.26E+02	[M+H]+	Tricin 4'-O-β-guaiacylglycerol	Flavone
pmb1270	8.52E+02	3.31E+02	4.04E+00	8.51E+02	[M+H]+	Tricin 4'-O-(β-guaiacylglyceryl) ether 5-O-hexosyl-O-hexoside	Flavone
pmb1312	6.89E+02	3.31E+02	4.43E+00	6.88E+02	[M+H]+	Tricin 4'-O-(β-guaiacylglyceryl) ether 7-O-hexoside	Flavone
pmb1466	5.11E+02	3.31E+02	6.38E+00	5.10E+02	[M+H]+	Tricin 4'-O-syringic acid	Flavone
pmb2850	3.29E+02	3.14E+02	5.73E+00	3.30E+02	[M-H]-	Tricin	Flavone
pmb2999	4.61E+02	2.99E+02	3.87E+00	4.62E+02	[M-H]-	Chrysoeriol 5-O-hexoside	Flavone
pmb3024	4.47E+02	3.27E+02	3.45E+00	4.48E+02	[M-H]-	Luteolin C-hexoside	Flavone
pmb3028	5.35E+02	4.91E+02	3.75E+00	5.36E+02	[M-H]-	Tricin O-sinapic acid	Flavone

pmb3032	5.61E+02	3.29E+02	5.69E+00	5.62E+02	[M-H]-	Tricin O-malonyl rhamnoside	Flavone
pmb3041	5.21E+02	3.29E+02	3.81E+00	5.22E+02	[M-H]-	Tricin O-saccharic acid	Flavone
pmb3042	4.91E+02	3.29E+02	4.00E+00	4.92E+02	[M-H]-	Tricin 5-O-hexoside	Flavone
pmb3047	6.57E+02	4.95E+02	4.50E+00	6.58E+02	[M-H]-	Tricin 4'-O-(syringyl alcohol) ether 5-O-hexoside	Flavone
pme0355	2.55E+02	1.37E+02	4.97E+00	2.54E+02	[M+H]+	Daidzein	Isoflavone
pme0364	3.01E+02	2.86E+02	5.77E+00	3.00E+02	[M+H]+	Chrysoeriol	Flavone
pme0377	2.73E+02	1.53E+02	5.59E+00	2.72E+02	[M+H]+	Naringenin	Flavanone
pme0379	2.71E+02	1.53E+02	5.63E+00	2.70E+02	[M+H]+	Apigenin	Flavone
pme0426	1.23E+02	1.08E+02	4.05E+00	1.24E+02	[M-H]-	4-Methylcatechol	Polyphenol
pme1502	3.15E+02	3.00E+02	7.23E+00	3.14E+02	[M+H]+	Kumatakenin	Flavonol
pme1518	4.03E+02	3.73E+02	7.06E+00	4.02E+02	[M+H]+	Nobiletin	Flavone

pme1550	3.73E+02	3.43E+02	7.54E+00	3.72E+02	[M+H]+	Tangeretin	Flavone
pme1568	2.85E+02	2.57E+02	5.08E+00	2.86E+02	[M-H]-	"Orobol (5,7,3',4'-tetrahydroxyisoflavone)"	Isoflavone
pme1578	2.71E+02	2.15E+02	5.52E+00	2.70E+02	[M+H]+	"Genistein (4',5,7-Trihydroxyisoflavone)"	Isoflavone
pme1580	2.87E+02	1.35E+02	5.05E+00	2.88E+02	[M-H]-	Eriodictyol	Flavanone
pme1588	3.15E+02	1.51E+02	5.85E+00	3.16E+02	[M-H]-	Isorhamnetin	Flavonol
pme1598	4.63E+02	3.01E+02	3.85E+00	4.64E+02	[M-H]-	Hesperetin 5-O-glucoside	Flavanone
pme1600	3.03E+02	1.67E+02	6.28E+00	3.02E+02	[M+H]+	7-O-Methyleriodictyol	Flavanone
pme1824	1.53E+02	1.09E+02	2.48E+00	1.54E+02	[M-H]-	Protocatechuic acid	Polyphenol
pme2482	1.39E+02	9.28E+01	3.03E+00	1.38E+02	[M+H]+	Protocatechuic aldehyde	Polyphenol
pme2954	3.03E+02	1.53E+02	5.13E+00	3.02E+02	[M+H]+	Quercetin	Flavonol

pme2957	2.71E+02	1.51E+02	5.57E+00	2.72E+02	[M-H]-	Naringenin chalcone	Chalcone
pme3137	2.39E+02	1.65E+02	7.73E+00	2.38E+02	[M+H]+	3-Hydroxyflavone	Flavone
pme3230	2.83E+02	2.68E+02	5.00E+00	2.84E+02	[M-H]-	Calycosin	Isoflavone
pme3282	2.73E+02	2.29E+02	3.40E+00	2.74E+02	[M-H]-	"Afzelechin (3,5,7,4'-Tetrahydroxyflavan)"	Flavanonol
pme3292	2.83E+02	2.68E+02	6.97E+00	2.84E+02	[M-H]-	Prunetin	Isoflavone
pme3464	2.85E+02	1.64E+02	6.98E+00	2.86E+02	[M-H]-	Isosakuranetin (4'-Methylnaringenin)	Flavanone
pme3473	2.71E+02	1.51E+02	5.59E+00	2.72E+02	[M-H]-	Butin	Flavanone
pmf0011	5.93E+02	4.73E+02	3.19E+00	5.94E+02	[M-H]-	"Apigenin 6,8-C-diglucoside"	Flavone
pmf0012	5.95E+02	4.57E+02	3.18E+00	5.94E+02	[M+H]+	"6,8-di-C-glucoside Apigenine"	Flavone
pmf0057	2.71E+02	1.51E+02	5.66E+00	2.72E+02	[M-H]-	"4,2',4',6'-Tetrahydroxychalcone	Chalcone

						"	
pmf0058	2.71E+02	1.51E+02	5.66E+00	2.72E+02	[M-H]-	"4',5,7-Trihydroxyflavanone"	Flavanone
pmf0179	6.23E+02	3.15E+02	4.04E+00	6.24E+02	[M-H]-	Narcissoside	Flavonol
pmf0204	4.65E+02	3.03E+02	3.73E+00	4.64E+02	[M+H]+	Hyperoside	Flavonol
pmf0208	4.65E+02	3.03E+02	3.90E+00	4.64E+02	[M+H]+	Isoquercitroside	Flavonol
pmf0375	4.79E+02	3.17E+02	4.18E+00	4.78E+02	[M+H]+	Isorhamnetin 3-O-glucoside	Flavonol
pmf0417	5.95E+02	2.87E+02	3.70E+00	5.96E+02	[M-H]-	Eriocitrin	Flavanone
pmf0548	5.63E+02	3.53E+02	3.34E+00	5.96E+02	[M-H]-	Schaftoside	Flavone
pmf0549	6.07E+02	2.99E+02	4.14E+00	6.08E+02	[M-H]-	Diosmin	Flavone
pmf0567	2.99E+02	2.11E+02	5.72E+00	3.00E+02	[M-H]-	Tectorigenin	Isoflavone
pma6499	5.09E+02	3.47E+02	3.41E+00	5.08E+02	[M+H]+	Limocitrin O-hexoside	Flavonol
pmb0277	3.31E+02	3.15E+02	5.74E+00	3.30E+02	[M+H]+	Tricin	Flavone

pmb0602	5.09E+02	3.47E+02	4.17E+00	5.08E+02	[M+H]+	Syringetin 7-O-hexoside	Flavonol
pmb0613	7.57E+02	5.77E+02	2.68E+00	7.56E+02	[M+H]+	Apigenin 6-C-hexosyl-8-C-hexosyl-O-hexoside	Flavone
pmb0644	4.49E+02	2.99E+02	3.45E+00	4.48E+02	[M+H]+	Luteolin C-hexoside	Flavone
pmb0674	6.85E+02	3.68E+02	4.10E+00	6.84E+02	[M+H]+	C-pentosyl apigenin O-salicyloyl hexoside	Flavone
pmb0711	6.11E+02	3.03E+02	3.70E+00	6.10E+02	[M+H]+	Quercetin 7-O-rutinoside	Flavonol
pmb1108	7.73E+02	5.93E+02	2.66E+00	7.72E+02	[M+H]+	Luteolin 6-C-hexoside 8-C-hexosyl-O-hexoside	Flavone
pmb3039	7.03E+02	3.29E+02	5.99E+00	7.04E+02	[M-H]-	Tricin O-oxalic acid O-coumaroyl shikimic acid	Flavone
pmb3043	6.37E+02	3.29E+02	4.07E+00	6.38E+02	[M-H]-	Tricin 5-O-rutinoside	Flavone

pmb3052	5.25E+02	3.29E+02	5.84E+00	5.26E+02	[M-H]-	Tricin 4'-O-β-guaiacylglycerol	Flavone
pmb3894	3.29E+02	2.29E+02	5.91E+00	3.30E+02	[M-H]-	Di-O-methylquercetin	Flavonol
pme0197	6.09E+02	3.01E+02	3.70E+00	6.10E+02	[M-H]-	Quercetin 3-O-rutinoside (Rutin)	Flavonol
pme1583	2.89E+02	1.53E+02	5.05E+00	2.88E+02	[M+H]+	Eriodictyol	Flavanone
pme2478	1.37E+02	1.08E+02	3.06E+00	1.38E+02	[M-H]-	Protocatechuic aldehyde	Polyphenol
pme2960	2.73E+02	1.53E+02	5.57E+00	2.72E+02	[M+H]+	Naringenin chalcone	Chalcone
pme3134	2.37E+02	2.09E+02	7.73E+00	2.38E+02	[M-H]-	3-Hydroxyflavone	Flavone
pmf0374	4.77E+02	3.14E+02	4.19E+00	4.78E+02	[M-H]-	Isorhamnetin 3-O-glucoside	Flavonol
pmf0550	6.09E+02	3.01E+02	4.15E+00	6.08E+02	[M+H]+	Diosmin	Flavone

Table S3 Qualitative determination of all flavonoids

Index	Compounds	Class	WL		WS		CL			CS_I			CS_ II			Flavonoids additive *in vitra* culture
			Diet	Feces	Diet	Feces	Diet	Feces	Plasma	Diet	Feces	Plasma	Diet	Feces	Plasma	
mws4091	Vitexin(Apigenin 8-C-glucoside)	Flavone	+	+	-	+	+	+	-	+	+	-	-	+	-	+
mws4092	Orientin(luteolin 8-C-glucoside)	Flavone	+	+	-	-	+	+	+	-	-	+	-	-	-	+
pma0214	methylQuercetin O-hexoside	Flavonol	+	+	+	+	+	+	-	+	+	-	+	+	-	+
pma0249	Selgin 5-O-hexoside	Flavone	+	+	-	+	+	+	-	-	-	-	-	-	-	+
pma0724	Naringenin C-hexoside	Flavanone	+	+	-	+	+	+	-	+	+	-	-	-	-	+
pma0787	Quercetin-3-(6"-malonyl)-Glucoside	Flavonol	-	+	-	-	-	+	-	-	-	-	-	-	-	-
pma0791	Naringenin O-malonylhexoside	Flavanone	+	+	+	+	+	+	-	+	+	-	+	-	-	-
pma0795	Tricetin O-malonylhexoside	Flavone	+	+	+	+	+	+	-	-	-	-	-	-	-	-

pma1108	Apigenin C-glucoside	Flavone	+	+	+	+	+	+	-	+	+	-	-	+	-	+
pma1116	Kaempferide	Flavonol	+	+	+	+	+	+	-	+	+	-	-	+	-	+
pma6218	O-methylnaringenin C-pentoside	Flavanone	+	+	-	-	+	+	-	-	+	-	-	+	-	-
pma6229	Eriodictyol C-hexoside	Flavanone	+	+	-	+	+	+	-	-	+	-	-	+	-	-
pma6371	"di-C,C-hexosyl-luteolin"	Flavone	+	+	+	-	+	+	-	-	+	-	-	-	-	+
pma6389	Ayanin	Flavonol	+	+	+	+	+	+	-	+	+	-	+	+	-	-
pma6499	Limocitrin O-hexoside	Flavonol	+	-	-	-	+	-	-	-	-	-	+	-	-	-
pma6520	Tricin 4'-O-(β-guaiacylglyceryl) ether-O-rutinoside	Flavone	+	+	-	-	+	+	-	-	+	-	-	-	-	-
pma6638	O-methylChrysoeriol 7-O-hexoside	Flavone	+	+	-	-	+	+	-	-	+	-	-	-	-	+
pmb0277	Tricin	Flavone	+	-	-	-	+	-	+	+	-	+	-	-	+	+

pmb0378	Luteolin O-feruloylhexoside	Flavone	+	+	-	-	+	+	-	-	+	-	-	-	-	-
pmb0565	Syringetin 3-O-hexoside	Flavonol	+	+	-	-	+	+	-	-	-	-	-	-	-	-
pmb0569	Syringetin 5-O-hexoside	Flavonol	+	+	+	+	+	+	-	+	-	-	+	+	-	-
pmb0576	Apigenin O-malonylhexoside	Flavone	+	+	+	+	+	+	-	+	+	-	+	+	-	-
pmb0595	Isorhamnetin 5-O-hexoside	Flavonol	+	+	+	+	+	+	-	+	+	-	+	+	-	+
pmb0602	Syringetin 7-O-hexoside	Flavonol	+	-	+	-	+	-	-	+	-	-	+	-	-	-
pmb0607	Chrysoeriol 7-O-hexoside	Flavone	+	+	-	+	+	+	-	+	+	-	-	+	-	+
pmb0608	Chrysoeriol O-malonylhexoside	Flavone	+	+	+	+	+	+	-	+	+	-	-	-	-	+
pmb0613	Apigenin 6-C-hexosyl-8-C-hexosyl-O-hexoside	Flavone	+	-	-	-	-	-	-	-	-	-	-	-	-	-
pmb0622	C-hexosyl-luteolin O-hexoside	Flavone	+	+	+	-	+	+	-	-	+	-	-	-	-	+
pmb0624	6-C-hexosyl-luteolin O-hexoside	Flavone	+	+	-	-	+	+	+	+	+	-	+	-	-	+

pmb0628	Eriodictiol C-hexosyl-O-hexoside	Flavanone	+	+	-	-	+	+	+	-	-	-	+	-	-	-
pmb0631	C-hexosyl-luteolin C-pentoside	Flavone	+	+	-	-	+	+	-	-	+	-	-	-	-	-
pmb0636	6-C-hexosyl luteolin O-pentoside	Flavone	+	+	-	-	+	+	-	-	+	-	-	-	-	-
pmb0644	Luteolin C-hexoside	Flavone	+	-	-	-	+	-	-	-	-	-	-	-	-	+
pmb0647	8-C-hexosyl-luteolin O-pentoside	Flavone	+	+	+	-	+	+	-	+	+	-	-	-	-	+
pmb0652	C-hexosyl-apigenin O-pentoside	Flavone	+	+	-	-	+	+	-	-	+	-	-	-	-	+
pmb0653	"di-C,C-hexosyl-apigenin"	Flavone	-	-	-	-	-	+	-	-	+	-	-	+	-	+
pmb0661	Chrysoeriol C-hexosyl-O-rhamnoside	Flavone	+	+	-	+	+	+	+	-	+	+	-	+	-	+
pmb0673	Apigenin 6-C-pentoside	Flavone	-	+	-	-	+	+	-	-	+	-	-	-	-	-
pmb0674	C-pentosyl apigenin O-salicyloyl hexoside	Flavone	-	-	-	-	-	-	-	-	-	-	-	-	-	-

pmb0681	Apigenin 8-C-pentoside	Flavone	+	+	-	-	+	+	-	+	+	-	+	-	-	+
pmb0686	Eriodictyol O-malonylhexoside	Flavanone	+	+	-	+	+	+	-	-	+	-	-	-	-	-
pmb0689	Chrysoeriol C-hexoside	Flavone	+	+	-	-	+	+	-	+	+	-	-	-	-	+
pmb0693	C-hexosyl-apigenin C-pentoside	Flavone	+	+	-	+	+	+	-	+	+	-	-	+	-	+
pmb0701	Chrysoeriol 8-C-hexoside	Flavone	+	+	-	+	+	+	-	-	+	-	-	+	-	-
pmb0711	Quercetin 7-O-rutinoside	Flavonol	-	-	+	-	+	-	-	-	-	-	+	-	-	+
pmb0712	Tricin 5-O-hexosyl-O-hexoside	Flavone	+	+	-	-	+	+	-	+	+	-	+	+	-	+
pmb0713	Tricin 7-O-hexosyl-O-hexoside	Flavone	+	+	+	+	+	+	-	+	+	-	+	+	-	+
pmb0714	Tricin 4'-O-(β-guaiacylglyceryl) ether 5-O-hexoside	Flavone	+	+	-	+	+	+	-	-	+	-	-	+	-	+
pmb0717	Tricin 5-O-β-guaiacylglycerol	Flavone	+	+	-	+	+	+	-	+	+	-	-	+	-	+

pmb0723	Tricin 4'-O-(β-guaiacylglyceryl) ether O-hexoside	Flavone	+	+	-	+	+	+	-	+	+	-	+	+	-	-
pmb0724	Tricin O-rhamnoside	Flavone	+	+	-	-	+	+	-	-	-	-	-	-	-	+
pmb0725	Tricin 7-O-feruloylhexoside	Flavone	+	+	-	+	+	+	-	-	+	-	-	-	-	-
pmb0733	Tricin 5-O-rutinoside	Flavone	-	+	-	+	-	+	-	-	+	-	-	+	-	-
pmb0735	Tricin O-glucuronic acid	Flavone	-	+	-	+	-	+	-	-	+	-	-	+	-	-
pmb0736	Tricin 7-O-hexoside	Flavone	+	+	-	-	+	+	-	+	+	-	-	+	-	+
pmb0738	Tricin O-sinapoylhexoside	Flavone	+	+	-	-	+	+	-	+	+	-	-	+	-	-
pmb0739	Tricin O-hexosyl-O-syringin alcohol	Flavone	+	+	-	-	+	+	-	-	-	-	-	-	-	-
pmb0743	Tricin 7-O-β-guaiacylglycerol	Flavone	+	+	-	-	+	+	-	-	-	-	-	-	-	-
pmb0744	Tricin O-phenylformic acid	Flavone	+	+	+	+	+	+	-	+	+	-	+	+	-	-

pmb0745	Tricin 4'-O-syringyl alcohol	Flavone	+	+	+	+	+	+	-	+	+	-	+	+	-	+
pmb0746	Tricin 4'-O-β-guaiacylglycerol	Flavone	-	+	-	+	-	+	+	-	+	-	-	+	-	-
pmb1108	Luteolin 6-C-hexoside 8-C-hexosyl-O-hexoside	Flavone	+	-	-	-	+	-	-	-	-	-	-	-	-	-
pmb1270	Tricin 4'-O-(β-guaiacylglyceryl) ether 5-O-hexosyl-O-hexoside	Flavone	+	+	-	-	+	+	-	-	-	-	-	-	-	-
pmb1312	Tricin 4'-O-(β-guaiacylglyceryl) ether 7-O-hexoside	Flavone	+	+	-	+	+	+	-	-	+	-	-	+	-	-
pmb1466	Tricin 4'-O-syringic acid	Flavone	+	+	-	+	+	+	-	-	+	-	-	+	-	+
pmb2850	Tricin	Flavone	-	+	-	+	-	+	-	-	+	-	-	+	-	-
pmb2999	Chrysoeriol 5-O-hexoside	Flavone	+	+	+	+	+	+	-	+	+	-	-	+	-	+
pmb3024	Luteolin C-hexoside	Flavone	-	+	-	-	-	+	+	-	-	+	-	-	-	-

pmb3028	Tricin O-sinapic acid	Flavone	+	+	+	+	+	+	-	+	+	-	+	+	-	-
pmb3032	Tricin O-malonyl rhamnoside	Flavone	-	+	-	+	-	+	-	-	+	-	-	+	-	-
pmb3039	Tricin O-oxalic acid O-coumaroyl shikimic acid	Flavone	+	-	-	-	+	-	-	+	-	-	-	-	-	-
pmb3041	Tricin O-saccharic acid	Flavone	+	+	+	+	+	+	-	+	+	-	+	+	-	-
pmb3042	Tricin 5-O-hexoside	Flavone	+	+	+	+	+	+	-	+	+	-	-	+	-	+
pmb3043	Tricin 5-O-rutinoside	Flavone	+	-	+	-	+	-	-	+	-	-	+	-	-	-
pmb3047	Tricin 4'-O-(syringyl alcohol) ether 5-O-hexoside	Flavone	+	+	-	+	+	+	-	-	+	-	-	-	-	-
pmb3052	Tricin 4'-O-β-guaiacylglycerol	Flavone	+	-	+	-	+	-	-	+	-	-	+	-	-	+
pmb3894	Di-O-methylquercetin	Flavonol	+	-	+	-	+	-	-	+	-	-	+	-	-	+
pme0197	Quercetin 3-O-rutinoside (Rutin)	Flavonol	+	-	+	-	+	-	-	+	-	-	+	-	-	+

pme0355	Daidzein	Isoflavone	+	+	-	-	+	+	-	-	+	-	-	+	-	+
pme0364	Chrysoeriol	Flavone	+	+	+	+	+	+	-	+	+	-	+	+	-	+
pme0377	Naringenin	Flavanone	+	+	+	+	+	+	-	+	+	-	+	+	-	+
pme0379	Apigenin	Flavone	+	+	+	+	+	+	-	+	+	-	-	+	-	+
pme0426	4-Methylcatechol	Polyphenol	-	+	-	-	-	+	-	-	+	-	-	-	-	-
pme1502	Kumatakenin	Flavonol	+	+	+	+	+	+	-	+	+	-	-	+	-	+
pme1518	Nobiletin	Flavone	+	+	+	+	+	+	+	+	+	+	+	+	+	+
pme1550	Tangeretin	Flavone	+	+	+	+	+	+	+	+	+	+	+	+	+	+
pme1568	"Orobol (5,7,3',4'-tetrahydroxyisoflavone)"	Isoflavone	+	+	-	-	+	+	-	-	-	-	-	-	-	-
pme1578	"Genistein (4',5,7-Trihydroxyisoflavone)"	Isoflavone	-	+	-	-	-	+	-	-	+	-	-	+	-	-

pme1580	Eriodictyol	Flavanone	-	+	-	+	-	+	-	-	+	-	-	+	-	-
pme1583	Eriodictyol	Flavanone	+	-	+	-	+	-	-	-	-	-	-	-	-	+
pme1588	Isorhamnetin(3'-Methylquercetin)	Flavonol	+	+	+	+	+	+	-	-	-	-	-	-	-	+
pme1598	Hesperetin 5-O-glucoside	Flavanone	+	+	+	+	+	+	-	-	+	-	-	+	-	+
pme1600	7-O-Methyleriodictyol	Flavanone	-	+	-	+	-	+	-	-	-	-	-	-	-	-
pme1824	Protocatechuic acid	Polyphenol	+	+	+	+	+	+	+	+	+	+	+	+	+	-
pme2478	Protocatechuic aldehyde	Polyphenol	+	-	+	-	+	-	-	+	-	-	+	-	-	-
pme2482	Protocatechuic aldehyde	Polyphenol	-	+	-	+	-	+	-	-	+	-	-	+	-	-
pme2954	Quercetin	Flavonol	-	+	-	-	-	+	-	-	+	-	-	-	-	-
pme2957	Naringenin chalcone	Chalcone	-	+	-	+	-	+	-	-	+	-	-	+	-	-
pme2960	Naringenin chalcone	Chalcone	-	-	-	-	-	-	-	-	-	-	-	-	-	-
pme3134	3’-Hydroxyflavone	Flavone	-	-	-	-	+	-	-	-	-	-	+	-	-	-

pme3137	3’-Hydroxyflavone	Flavone	-	-	-	+	-	+	-	-	+	-	-	-	-	-
pme3230	Calycosin	Isoflavone	-	-	-	-	-	+	-	-	+	-	-	+	-	-
pme3282	"Afzelechin (3,5,7,4'-Tetrahydroxyflavan)"	Flavanonol	-	+	+	+	+	+	+	+	+	+	+	+	+	-
pme3292	Prunetin	Isoflavone	-	+	-	+	-	+	-	-	+	-	-	+	-	-
pme3464	Isosakuranetin (4'-Methylnaringenin)	Flavanone	+	+	+	+	+	+	-	+	-	-	-	-	-	-
pme3473	Butin	Flavanone	+	+	+	+	+	+	-	+	+	-	+	+	-	+
pmf0011	"Apigenin 6,8-C-diglucoside"	Flavone	+	+	+	+	+	+	-	+	+	-	+	+	-	+
pmf0012	"Apigenin 6,8-C-diglucoside"	Flavone	+	+	+	+	+	+	-	-	+	-	-	+	-	-
pmf0057	Naringenin chalcone	Chalcone	+	+	+	+	+	+	-	+	+	-	+	+	-	-
pmf0058	Naringenin	Flavanone	+	+	+	+	+	+	-	+	+	-	+	+	-	+

pmf0179	Narcissoside(3'-Methylquercetin 3-O-rutinoside)	Flavonol	+	+	+	+	+	+	-	+	+	-	+	+	-	+
pmf0204	Hyperoside(Quercetin 3-D-galactoside)	Flavonol	+	-	+	+	+	+	-	+	+	-	+	+	-	+
pmf0208	Isoquercitroside	Flavonol	+	-	+	+	+	+	-	+	+	-	-	+	-	+
pmf0374	Isorhamnetin 3-O-glucoside	Flavonol	+	-	+	-	+	-	-	-	-	-	+	-	-	+
pmf0375	Isorhamnetin 3-O-glucoside	Flavonol	-	+	-	+	-	+	-	-	+	-	-	+	-	-
pmf0417	Eriocitrin	Flavanone	+	+	+	-	+	+	-	-	+	-	-	-	-	+
pmf0548	Schaftoside	Flavone	+	+	+	+	+	+	+	+	+	+	+	+	+	+
pmf0549	Diosmin	Flavone	-	+	-	+	-	+	-	-	+	-	-	+	-	-
pmf0550	Diosmin	Flavone	+	-	+	-	+	-	-	+	-	-	-	-	-	+
pmf0567	Tectorigenin	Isoflavone	+	+	-	+	+	+	-	+	+	-	-	+	-	+

Notes: “+” and “-” indicate the positive and negative detection, respectively.

Table S4 Monomers with different proportion in diet and feces.

Group	N	Trend	Index	Class	Mean percentage in diet	Mean percentage in feces	Method of statistics	Statistics F or W	*P*	FDR
WL	74	Diet > Feces (n=18); Diet < Feces (n=0)	pme0355	Isoflavone	4.0E-05	7.9E-06	anova	1.50E+01	1.94E-03	1.03E-02
			pmb3047	Flavone	8.0E-04	5.0E-04	anova	1.23E+01	3.87E-03	1.79E-02
			pme1598	Flavanone	9.3E-04	2.9E-04	anova	1.79E+01	9.76E-04	6.02E-03
			pmf0179	Flavonol	1.4E-03	6.9E-04	anova	1.17E+01	4.58E-03	1.99E-02
			pmb0744	Flavone	2.0E-03	9.4E-04	anova	1.07E+02	1.20E-07	1.77E-06
			pma6389	Flavonol	2.2E-03	1.0E-03	anova	2.70E+01	1.72E-04	1.16E-03
			pmb2999	Flavone	3.3E-03	9.3E-04	anova	3.81E+01	3.35E-05	2.48E-04
			pmb0661	Flavone	4.2E-03	1.1E-03	anova	1.67E+02	8.51E-09	2.10E-07

			pmb0628	Flavanone	6.3E-03	3.0E-03	anova	1.11E+01	5.38E-03	2.13E-02
			pmb0736	Flavone	8.1E-03	1.7E-03	anova	8.08E+01	6.09E-07	7.52E-06
			pmf0011	Flavone	2.2E-02	1.4E-02	anova	1.57E+01	1.64E-03	9.31E-03
			pmb0576	Flavone	1.3E-02	3.4E-03	anova	5.17E+01	7.03E-06	6.50E-05
			pma0249	Flavone	2.3E-02	5.6E-03	anova	1.54E+02	1.40E-08	2.60E-07
			pma0795	Flavone	2.2E-02	2.8E-03	anova	1.80E+02	5.34E-09	1.98E-07
			pmb3042	Flavone	3.6E-02	1.2E-02	anova	5.21E+01	6.78E-06	6.50E-05
			pmb0689	Flavone	3.0E-02	1.1E-03	anova	6.32E+02	2.09E-12	1.55E-10
			pma1116	Flavonol	7.9E-02	3.6E-02	anova	1.42E+01	2.33E-03	1.15E-02
			pma0791	Flavanone	5.8E-02	1.1E-02	anova	4.73E+01	1.13E-05	9.28E-05
WS	34	Diet > Feces (n=4)	pme1518	Flavone	1.5E-03	6.2E-04	anova	1.04E+01	8.16E-03	3.03E-02
			pma0791	Flavanone	8.3E-03	1.9E-03	anova	1.40E+01	3.29E-03	3.03E-02

		Diet < Feces	pmb0576	Flavone	1.4E-02	3.7E-03	anova	2.04E+01	8.75E-04	1.49E-02
		(n=0)	pmf0208	Flavonol	7.7E-02	1.2E-02	anova	2.19E+02	1.32E-08	4.49E-07
CL	78	Diet < Feces (n=9)	pmb0607	Flavone	2.2E-03	5.8E-02	wilcox	0.00E+00	5.56E-03	1.55E-02
			pmb1312	Flavone	1.5E-03	4.3E-02	wilcox	0.00E+00	5.56E-03	1.55E-02
			pme0377	Flavanone	6.7E-04	3.7E-02	wilcox	0.00E+00	5.56E-03	1.55E-02
			pmb0693	Flavone	9.9E-04	3.6E-02	wilcox	0.00E+00	5.56E-03	1.55E-02
			pmb0701	Flavone	4.2E-04	2.3E-02	wilcox	0.00E+00	5.56E-03	1.55E-02
			pme0379	Flavone	3.6E-04	1.8E-02	wilcox	0.00E+00	5.56E-03	1.55E-02
			pme3473	Flavanone	2.7E-04	1.6E-02	wilcox	0.00E+00	5.56E-03	1.55E-02
			pme3282	Flavanonol	5.6E-05	2.2E-03	wilcox	0.00E+00	5.56E-03	1.55E-02
			pmf0567	Isoflavone	5.5E-05	3.7E-04	wilcox	0.00E+00	5.56E-03	1.55E-02
		Diet > Feces	pmb0743	Flavone	2.8E-04	7.7E-05	anova	5.25E+01	5.60E-08	4.03E-07

		(n=20)	pmb3028	Flavone	4.7E-04	6.5E-05	anova	5.48E+01	3.69E-08	3.19E-07
			pme1588	Flavonol	1.1E-03	6.8E-05	anova	1.01E+03	4.57E-24	1.78E-22
			pmb3041	Flavone	1.9E-03	2.1E-04	anova	1.71E+02	1.06E-13	2.07E-12
			pmb0636	Flavone	2.7E-03	9.2E-04	anova	3.26E+01	3.55E-06	2.13E-05
			pma6389	Flavonol	2.6E-03	5.5E-04	anova	5.24E+01	5.68E-08	4.03E-07
			pmb0631	Flavone	4.9E-03	2.8E-03	wilcox	8.20E+01	8.31E-03	2.13E-02
			pmb2999	Flavone	3.9E-03	5.8E-04	anova	1.24E+02	5.29E-12	5.89E-11
			pmb0628	Flavanone	6.2E-03	6.7E-04	anova	2.68E+02	3.39E-16	8.82E-15
			pma0249	Flavone	8.0E-03	1.6E-03	anova	2.85E+01	1.00E-05	5.20E-05
			pma0795	Flavone	1.3E-02	1.2E-03	anova	1.51E+02	4.90E-13	7.64E-12
			pmb0647	Flavone	1.6E-02	3.1E-03	anova	6.11E+01	1.27E-08	1.24E-07
			mws4092	Flavone	1.8E-02	5.8E-04	anova	2.97E+03	9.16E-31	7.14E-29

			pmb0608	Flavone	4.5E-02	5.8E-03	wilcox	8.40E+01	5.56E-03	1.55E-02
			pmb0622	Flavone	6.2E-02	1.7E-02	anova	3.12E+01	5.00E-06	2.79E-05
			pma6371	Flavone	6.8E-02	1.7E-02	wilcox	8.40E+01	5.56E-03	1.55E-02
			pmb3042	Flavone	6.2E-02	9.0E-03	anova	1.37E+02	1.72E-12	2.24E-11
			pma1116	Flavonol	9.8E-02	2.2E-02	anova	4.77E+01	1.36E-07	8.84E-07
			pmf0011	Flavone	9.9E-02	2.1E-02	wilcox	8.40E+01	5.56E-03	1.55E-02
			pmb0624	Flavone	1.2E-01	1.8E-02	wilcox	8.40E+01	5.56E-03	1.55E-02
CS_I	43	Diet > Feces (n=15) Diet < Feces (n=0)	pmb0681	Flavone	6.4E-04	8.8E-05	anova	2.00E+01	7.60E-04	3.63E-03
			pma0791	Flavanone	6.6E-04	9.1E-05	anova	1.18E+01	4.92E-03	1.47E-02
			pmb0689	Flavone	1.0E-03	2.9E-04	anova	1.34E+01	3.30E-03	1.09E-02
			pmb2999	Flavone	2.0E-03	4.7E-04	anova	1.17E+01	5.14E-03	1.47E-02
			pmb3041	Flavone	2.9E-03	3.2E-04	anova	1.10E+02	2.18E-07	2.35E-06

			pmb0647	Flavone	4.0E-03	2.1E-04	anova	1.23E+02	1.18E-07	1.69E-06
			pmb0608	Flavone	4.9E-03	1.8E-04	anova	2.39E+02	2.78E-09	5.98E-08
			pma6389	Flavonol	5.7E-03	3.0E-04	anova	3.02E+02	7.11E-10	3.06E-08
			pma0214	Flavonol	8.0E-03	1.7E-03	anova	4.79E+01	1.61E-05	1.15E-04
			pmb3028	Flavone	9.4E-03	1.6E-03	anova	3.84E+01	4.61E-05	2.48E-04
			pmf0179	Flavonol	1.1E-02	3.3E-03	anova	1.90E+01	9.30E-04	4.00E-03
			pmb0595	Flavonol	1.3E-02	2.6E-03	anova	4.39E+01	2.43E-05	1.50E-04
			pma1116	Flavonol	1.8E-02	4.0E-03	anova	1.47E+01	2.39E-03	9.34E-03
			pmb3042	Flavone	2.5E-02	2.6E-03	anova	6.35E+01	3.91E-06	3.36E-05
			pme1824	Polyphenol	4.6E-01	1.0E-01	anova	1.38E+01	2.96E-03	1.06E-02
CS_II	23	Diet > Feces	pme1550	Flavone	5.5E-03	2.8E-03	anova	1.35E+01	3.68E-03	1.41E-02
		(n=7)	pmb3041	Flavone	3.4E-03	3.5E-04	anova	2.53E+02	6.15E-09	1.05E-07

		Diet < Feces (n=0)	pma6389	Flavonol	3.8E-03	5.0E-04	anova	2.35E+02	9.15E-09	1.05E-07
			pme1518	Flavone	6.2E-03	2.6E-03	anova	2.43E+01	4.51E-04	2.08E-03
			pmf0204	Flavonol	2.5E-02	5.5E-03	anova	7.09E+01	4.00E-06	3.07E-05
			pma0214	Flavonol	1.8E-01	7.5E-02	anova	1.09E+01	6.97E-03	2.29E-02
			pme1824	Polyphenol	1.4E-01	2.7E-02	anova	4.04E+01	5.39E-05	3.10E-04

N: The number of shared flavonoids in both diet and corresponding feces; Trend: The changing trend from diet to the feces (the number of flavonoids); anova: one-way analysis of variance; wilcox: Wilcoxon rank sum test.

Table S5 Sample information for metagenomic analysis and assembly information.

Sample_ID	Original name	Group	Data source	Clean base (bp)	Contigs	Contigs bases (bp)	N50 (bp)	N90 (bp)	Max (bp)	Min (bp)
1	A074	WL	published data	1.92E+09	3.14E+03	9.09E+06	3.50E+04	6.24E+02	3.88E+05	3.00E+02
2	AM120105	WL	published data	4.87E+09	3.41E+04	2.69E+07	8.59E+02	3.78E+02	9.06E+04	3.00E+02
3	AM120401	WL	published data	4.69E+09	2.51E+04	2.62E+07	1.56E+03	4.14E+02	1.25E+05	3.00E+02
4	AM120405	WL	published data	5.94E+09	6.53E+04	5.95E+07	1.13E+03	3.89E+02	3.80E+05	3.00E+02
5	AM120406	WL	published data	6.53E+09	3.38E+04	3.71E+07	1.63E+03	4.28E+02	1.11E+05	3.00E+02
6	AM120506	WS	published data	8.06E+09	5.23E+04	6.78E+07	2.91E+03	4.47E+02	2.20E+05	3.00E+02
7	AM120701	WS	published data	6.93E+09	5.41E+04	9.19E+07	4.10E+03	5.51E+02	2.59E+05	3.00E+02
8	AM120806	WS	published data	8.11E+09	3.22E+04	3.50E+07	1.76E+03	4.12E+02	4.95E+05	3.00E+02
9	AM1208X1	WS	published data	7.44E+09	2.21E+04	2.18E+07	1.38E+03	4.02E+02	1.15E+05	3.00E+02

10	AM130101	WL	published data	2.71E+09	1.91E+04	2.20E+07	2.09E+03	4.19E+02	1.63E+05	3.00E+02
11	AM130104	WL	published data	4.79E+09	2.50E+04	3.17E+07	2.79E+03	4.36E+02	2.62E+05	3.00E+02
12	AM130105	WL	published data	6.53E+09	4.99E+04	3.56E+07	7.56E+02	3.75E+02	9.35E+04	3.00E+02
13	AM130301	WL	published data	6.05E+09	1.73E+04	2.35E+07	3.17E+03	4.54E+02	7.57E+04	3.00E+02
14	AM130303	WL	published data	4.33E+09	2.11E+04	1.85E+07	1.05E+03	3.92E+02	2.40E+05	3.00E+02
15	AM130305	WL	published data	2.89E+09	2.09E+04	2.32E+07	2.00E+03	4.09E+02	6.03E+04	3.00E+02
16	AM130503	WS	published data	7.90E+09	5.97E+04	7.71E+07	2.41E+03	4.63E+02	2.50E+05	3.00E+02
17	AM130505	WS	published data	6.02E+09	3.18E+04	3.26E+07	1.70E+03	3.85E+02	1.90E+05	3.00E+02
18	AM130506	WS	published data	6.22E+09	1.15E+04	1.41E+07	1.96E+03	4.68E+02	5.68E+05	3.00E+02
19	AM130701	WS	published data	5.63E+09	4.76E+04	7.08E+07	4.19E+03	4.60E+02	2.02E+05	3.00E+02
20	AM130703	WS	published data	6.21E+09	1.61E+04	2.62E+07	6.01E+03	5.03E+02	4.81E+05	3.00E+02
21	AM130706	WS	published data	6.86E+09	3.42E+04	5.70E+07	4.67E+03	5.20E+02	2.48E+05	3.00E+02

22	AM1307X1	WS	published data	6.08E+09	3.09E+04	4.05E+07	2.44E+03	4.66E+02	4.20E+05	3.00E+02
23	AM130801	WS	published data	6.41E+09	3.55E+04	3.66E+07	1.43E+03	4.22E+02	2.70E+05	3.00E+02
24	AM130803	WS	published data	7.10E+09	3.29E+04	4.91E+07	3.53E+03	4.93E+02	4.70E+05	3.00E+02
25	AM130806	WS	published data	8.18E+09	1.53E+04	3.57E+07	1.36E+04	6.17E+02	4.64E+05	3.00E+02
26	AM1308X1	WS	published data	6.80E+09	4.77E+04	4.72E+07	1.42E+03	4.03E+02	1.43E+05	3.00E+02
27	WL1	WL	This study	1.82E+10	7.05E+04	7.09E+07	1.61E+03	3.82E+02	2.14E+05	3.00E+02
28	WL3	WL	This study	1.50E+10	6.86E+04	7.04E+07	1.62E+03	3.86E+02	1.90E+05	3.00E+02
29	WL4	WL	This study	1.51E+10	6.62E+04	6.57E+07	1.53E+03	3.83E+02	1.15E+05	3.00E+02
30	WL5	WL	This study	1.48E+10	7.30E+04	7.20E+07	1.49E+03	3.81E+02	3.39E+05	3.00E+02
31	WL9	WL	This study	1.53E+10	6.87E+04	7.53E+07	1.97E+03	3.92E+02	3.37E+05	3.00E+02
32	WL10	WL	This study	1.85E+10	4.41E+04	5.65E+07	2.67E+03	4.33E+02	1.50E+05	3.00E+02
33	WL11	WL	This study	1.81E+10	7.43E+04	8.19E+07	2.02E+03	3.95E+02	1.76E+05	3.00E+02

34	WL12	WL	This study	1.64E+10	5.25E+04	5.86E+07	2.04E+03	3.94E+02	1.76E+05	3.00E+02
35	WL15	WL	This study	1.48E+10	6.79E+04	6.58E+07	1.47E+03	3.75E+02	4.64E+05	3.00E+02
36	WL16	WL	This study	1.31E+10	4.97E+04	5.30E+07	1.74E+03	3.96E+02	1.54E+05	3.00E+02
37	WL17	WL	This study	1.65E+10	7.81E+04	9.61E+07	2.66E+03	4.11E+02	3.15E+05	3.00E+02
38	WL18	WL	This study	1.65E+10	4.52E+04	5.10E+07	1.94E+03	4.05E+02	4.23E+05	3.00E+02
39	WL20	WL	This study	1.34E+10	5.83E+04	5.97E+07	1.57E+03	3.90E+02	2.24E+05	3.00E+02
40	WL22	WL	This study	1.49E+10	8.53E+04	9.42E+07	1.87E+03	3.95E+02	2.58E+05	3.00E+02
41	WL24	WL	This study	1.67E+10	1.02E+05	8.98E+07	1.16E+03	3.72E+02	2.79E+05	3.00E+02
42	WL25	WL	This study	1.67E+10	1.38E+05	1.15E+08	1.06E+03	3.61E+02	2.14E+05	3.00E+02
43	WL27	WL	This study	1.27E+10	3.10E+04	4.12E+07	3.20E+03	4.29E+02	2.38E+05	3.00E+02
44	WS1	WS	This study	1.64E+10	1.40E+05	1.46E+08	1.70E+03	3.91E+02	2.72E+05	3.00E+02
45	WS2	WS	This study	1.59E+10	8.00E+04	8.26E+07	2.11E+03	3.70E+02	1.73E+05	3.00E+02

46	WS3	WS	This study	1.37E+10	9.14E+04	9.12E+07	1.98E+03	3.58E+02	3.27E+05	3.00E+02
47	WS4	WS	This study	1.67E+10	8.35E+04	7.87E+07	1.48E+03	3.66E+02	4.20E+05	3.00E+02
48	WS5	WS	This study	1.67E+10	3.85E+05	2.16E+08	5.06E+02	3.25E+02	1.33E+05	3.00E+02
49	WS6	WS	This study	1.59E+10	6.16E+04	5.70E+07	1.33E+03	3.74E+02	7.41E+04	3.00E+02
50	WS8	WS	This study	2.02E+10	1.71E+05	1.26E+08	7.78E+02	3.39E+02	1.35E+05	3.00E+02
51	WS7	WS	This study	1.39E+10	5.08E+04	6.18E+07	2.42E+03	4.17E+02	5.61E+05	3.00E+02
52	WS9	WS	This study	1.46E+10	6.35E+04	5.31E+07	1.00E+03	3.45E+02	5.78E+05	3.00E+02
53	CS13	CS	This study	1.51E+10	3.20E+05	2.86E+08	1.18E+03	3.79E+02	1.17E+05	3.00E+02
54	CS14	CS	This study	1.42E+10	1.52E+05	1.27E+08	1.03E+03	3.62E+02	1.24E+05	3.00E+02
55	CS15	CS	This study	1.40E+10	1.12E+05	1.04E+08	1.27E+03	3.75E+02	5.00E+05	3.00E+02
56	CS16	CS	This study	1.67E+10	1.23E+05	1.10E+08	1.22E+03	3.69E+02	2.62E+05	3.00E+02
57	CS17	CS	This study	1.73E+10	3.49E+05	2.95E+08	1.05E+03	3.73E+02	2.76E+05	3.00E+02

58	CS18	CS	This study	1.65E+10	2.40E+05	2.04E+08	1.06E+03	3.73E+02	2.56E+05	3.00E+02
59	CS19	CS	This study	1.40E+10	3.03E+05	2.53E+08	1.01E+03	3.72E+02	3.07E+05	3.00E+02
60	CS20	CS	This study	1.58E+10	2.49E+05	2.27E+08	1.21E+03	3.77E+02	2.36E+05	3.00E+02
61	CS21	CS	This study	1.51E+10	3.36E+05	2.83E+08	1.03E+03	3.72E+02	2.07E+05	3.00E+02
62	CS22	CS	This study	1.48E+10	2.42E+05	2.11E+08	1.13E+03	3.74E+02	1.19E+05	3.00E+02
63	CS23	CS	This study	1.44E+10	3.98E+05	3.13E+08	9.39E+02	3.63E+02	1.99E+05	3.00E+02
64	CS25	CS	This study	1.50E+10	1.14E+05	1.10E+08	1.53E+03	3.71E+02	2.23E+05	3.00E+02
65	CS26	CS	This study	1.62E+10	3.37E+05	3.11E+08	1.27E+03	3.80E+02	2.41E+05	3.00E+02
66	CS28	CS	This study	1.43E+10	1.93E+05	1.60E+08	1.03E+03	3.67E+02	1.23E+05	3.00E+02
67	CS29	CS	This study	1.46E+10	2.34E+05	2.00E+08	1.10E+03	3.65E+02	2.26E+05	3.00E+02
68	CS30	CS	This study	1.57E+10	6.37E+04	7.86E+07	2.74E+03	4.17E+02	1.46E+05	3.00E+02
69	CS31	CS	This study	1.52E+10	2.95E+05	2.73E+08	1.27E+03	3.82E+02	1.74E+05	3.00E+02

70	CS32	CS	This study	1.62E+10	3.25E+05	3.02E+08	1.27E+03	3.79E+02	3.13E+05	3.00E+02
71	CS33	CS	This study	1.53E+10	2.08E+05	1.98E+08	1.37E+03	3.84E+02	1.69E+05	3.00E+02
72	CS34	CS	This study	1.50E+10	2.46E+05	2.12E+08	1.10E+03	3.72E+02	2.58E+05	3.00E+02
73	CS35	CS	This study	1.46E+10	2.01E+05	1.73E+08	1.09E+03	3.73E+02	2.57E+05	3.00E+02
74	CT1		This study	1.39E+10	1.79E+04	1.73E+07	1.54E+03	3.67E+02	1.25E+05	3.00E+02
75	CT2		This study	1.38E+10	4.90E+04	5.12E+07	1.68E+03	3.99E+02	7.72E+04	3.00E+02
76	CT3		This study	1.58E+10	8.11E+04	5.76E+07	7.66E+02	3.34E+02	1.46E+05	3.00E+02
77	CT4		This study	1.45E+10	9.11E+04	9.56E+07	1.68E+03	3.89E+02	2.35E+05	3.00E+02
78	CT5		This study	1.49E+10	6.94E+04	6.61E+07	1.42E+03	3.71E+02	3.23E+05	3.00E+02
79	CT6		This study	1.43E+10	8.87E+04	8.39E+07	1.31E+03	3.74E+02	2.58E+05	3.00E+02
80	CT7		This study	1.49E+10	1.29E+05	1.14E+08	1.11E+03	3.73E+02	2.22E+05	3.00E+02
81	CT8		This study	1.51E+10	1.40E+05	1.27E+08	1.17E+03	3.83E+02	2.52E+05	3.00E+02

82	CT9		This study	1.63E+10	5.51E+04	7.28E+07	4.12E+03	4.08E+02	4.05E+05	3.00E+02
83	CT10		This study	1.32E+10	5.03E+04	4.77E+07	1.32E+03	3.74E+02	4.16E+05	3.00E+02
84	CL1	CL	This study	1.38E+10	1.12E+05	9.37E+07	1.05E+03	3.60E+02	1.25E+05	3.00E+02
85	CL2	CL	This study	1.51E+10	1.03E+05	9.06E+07	1.18E+03	3.67E+02	2.52E+05	3.00E+02
86	CL3	CL	This study	1.50E+10	1.08E+05	9.10E+07	1.11E+03	3.61E+02	1.38E+05	3.00E+02
87	CL4	CL	This study	1.52E+10	8.48E+04	7.74E+07	1.36E+03	3.62E+02	1.40E+05	3.00E+02
88	CL5	CL	This study	1.49E+10	7.30E+04	6.67E+07	1.30E+03	3.62E+02	1.68E+05	3.00E+02
89	CL7	CL	This study	1.40E+10	1.63E+04	2.11E+07	2.80E+03	4.37E+02	1.22E+05	3.00E+02
90	CL8	CL	This study	1.84E+10	8.22E+04	8.07E+07	1.53E+03	3.77E+02	4.13E+05	3.00E+02
91	CL9	CL	This study	1.37E+10	9.47E+04	8.29E+07	1.16E+03	3.67E+02	1.03E+05	3.00E+02
92	CL10	CL	This study	1.40E+10	6.81E+04	6.18E+07	1.30E+03	3.67E+02	1.09E+05	3.00E+02
93	CL11	CL	This study	1.36E+10	7.78E+04	6.94E+07	1.19E+03	3.72E+02	8.89E+05	3.00E+02

94	CL12	CL	This study	1.51E+10	7.51E+04	6.24E+07	1.05E+03	3.59E+02	1.11E+05	3.00E+02
95	CL13	CL	This study	1.48E+10	7.33E+04	6.87E+07	1.34E+03	3.79E+02	7.54E+04	3.00E+02
96	CL14	CL	This study	1.33E+10	7.92E+04	6.98E+07	1.19E+03	3.67E+02	1.12E+05	3.00E+02
97	CL15	CL	This study	1.57E+10	4.77E+04	4.76E+07	1.46E+03	3.87E+02	2.24E+05	3.00E+02
98	CL16	CL	This study	1.59E+10	5.26E+04	5.35E+07	1.62E+03	3.85E+02	2.39E+05	3.00E+02
99	CL17	CL	This study	1.34E+10	7.72E+04	7.47E+07	1.44E+03	3.76E+02	9.26E+05	3.00E+02
100	CL18	CL	This study	1.83E+10	7.38E+04	5.78E+07	9.22E+02	3.61E+02	1.46E+05	3.00E+02
101	CL21	CL	This study	1.39E+10	3.95E+04	3.71E+07	1.44E+03	3.71E+02	1.68E+05	3.00E+02
102	CL22	CL	This study	1.53E+10	7.50E+04	6.95E+07	1.31E+03	3.79E+02	6.44E+04	3.00E+02
103	CL23	CL	This study	1.48E+10	7.62E+04	6.94E+07	1.31E+03	3.66E+02	1.89E+05	3.00E+02
104	CL24	CL	This study	1.48E+10	9.06E+04	8.61E+07	1.49E+03	3.60E+02	3.44E+05	3.00E+02
105	CL25	CL	This study	1.50E+10	5.09E+04	4.82E+07	1.36E+03	3.79E+02	2.25E+05	3.00E+02

106	CL26	CL	This study	1.56E+10	8.87E+04	8.03E+07	1.27E+03	3.70E+02	1.69E+05	3.00E+02
107	CL27	CL	This study	1.47E+10	7.56E+04	7.02E+07	1.33E+03	3.74E+02	1.25E+05	3.00E+02
108	CL28	CL	This study	1.63E+10	1.09E+05	8.43E+07	1.02E+03	3.37E+02	2.45E+05	3.00E+02
109	CL29	CL	This study	1.52E+10	1.01E+05	9.15E+07	1.27E+03	3.69E+02	2.33E+05	3.00E+02
110	CL30	CL	This study	1.60E+10	6.21E+04	5.14E+07	1.07E+03	3.57E+02	1.07E+05	3.00E+02
111	19		published data	1.05E+10	9.82E+04	6.93E+07	7.91E+02	3.57E+02	4.90E+04	3.00E+02
112	20131124-ZX-F03		published data	9.37E+09	1.05E+05	8.29E+07	9.32E+02	3.54E+02	1.12E+05	3.00E+02
113	20140128-zx-F03		published data	1.16E+10	3.48E+04	3.39E+07	1.53E+03	3.85E+02	7.01E+04	3.00E+02
114	23		published data	7.76E+09	3.27E+05	2.18E+08	6.96E+02	3.49E+02	5.86E+04	3.00E+02
115	31		published data	7.07E+09	1.08E+05	6.06E+07	5.25E+02	3.31E+02	5.27E+04	3.00E+02
116	34		published data	7.78E+09	4.25E+05	2.37E+08	5.46E+02	3.38E+02	3.62E+04	3.00E+02
117	50		published data	7.12E+09	1.51E+05	8.21E+07	5.09E+02	3.30E+02	5.73E+04	3.00E+02

118	9		published data	8.51E+09	1.30E+04	1.18E+07	1.23E+03	3.59E+02	2.60E+05	3.00E+02
119	CDXM18		published data	8.48E+09	9.89E+04	7.47E+07	8.27E+02	3.58E+02	1.64E+05	3.00E+02
120	CDXM27		published data	8.23E+09	1.84E+05	1.48E+08	9.39E+02	3.67E+02	5.92E+04	3.00E+02
121	CDXM39		published data	9.79E+09	1.31E+05	1.03E+08	9.15E+02	3.69E+02	9.56E+04	3.00E+02
122	CDXM40		published data	1.21E+10	7.09E+04	6.43E+07	1.27E+03	3.81E+02	9.28E+04	3.00E+02
123	LX3		published data	2.33E+09	4.31E+04	4.71E+07	1.67E+03	4.32E+02	9.66E+04	3.00E+02
124	LZP1		published data	1.62E+09	2.14E+04	3.60E+07	3.75E+03	5.65E+02	3.17E+05	3.00E+02
125	TT1		published data	5.88E+08	9.15E+03	1.02E+07	3.40E+03	4.00E+02	1.14E+05	3.00E+02
126	TT2		published data	4.26E+09	3.83E+04	5.58E+07	3.14E+03	5.00E+02	2.21E+05	3.00E+02
127	CD.m71.110914		published data	6.67E+09	1.18E+04	2.84E+07	9.81E+03	6.96E+02	2.32E+05	3.00E+02
128	CD.m77.111001		published data	2.79E+09	1.30E+04	1.89E+07	7.19E+03	4.45E+02	1.55E+05	3.00E+02
129	CD.m121.111017		published data	5.70E+09	1.87E+04	2.88E+07	6.50E+03	4.47E+02	3.05E+05	3.00E+02

130	CD.70.130117		published data	7.14E+09	3.76E+04	3.59E+07	1.39E+03	3.95E+02	6.79E+04	3.00E+02
131	CD.73.130415		published data	5.02E+09	5.57E+04	5.09E+07	1.27E+03	3.80E+02	5.50E+04	3.00E+02
132	CX.m1.110827		published data	7.17E+09	3.33E+03	1.40E+07	6.89E+04	1.11E+03	2.34E+05	3.00E+02
133	CX.m18.110917		published data	3.85E+09	1.51E+04	2.08E+07	7.11E+03	4.30E+02	3.63E+05	3.00E+02
134	CX.m31.111104		published data	5.42E+09	2.23E+04	2.77E+07	5.69E+03	4.09E+02	2.58E+05	3.00E+02
135	CX.39.130208		published data	5.10E+09	2.81E+04	2.10E+07	8.26E+02	3.59E+02	8.33E+04	3.00E+02
136	CX.6.121213		published data	7.43E+09	1.44E+05	1.08E+08	7.75E+02	3.61E+02	4.59E+05	3.00E+02
137	EQ.m114.110825		published data	4.67E+09	8.27E+03	1.42E+07	1.59E+04	4.75E+02	4.06E+05	3.00E+02
138	EQ.m164.111020		published data	4.51E+09	2.68E+04	2.89E+07	1.87E+03	4.00E+02	2.21E+05	3.00E+02
139	EQ.m168.111104		published data	1.98E+09	4.61E+04	4.32E+07	1.39E+03	3.74E+02	2.32E+05	3.00E+02
140	EQ.118.130124		published data	7.77E+09	4.62E+04	4.38E+07	1.28E+03	3.96E+02	2.27E+05	3.00E+02
141	EQ.128.130507		published data	5.34E+09	9.21E+04	8.79E+07	1.38E+03	3.93E+02	1.42E+05	3.00E+02

142	HH.m275.080925		published data	3.66E+09	2.60E+04	2.63E+07	2.02E+03	3.83E+02	2.99E+05	3.00E+02
143	HH.m278.081017		published data	2.73E+09	2.50E+04	3.52E+07	6.42E+03	4.26E+02	2.51E+05	3.00E+02
144	HH.186.090510		published data	4.78E+09	5.53E+04	5.78E+07	1.58E+03	4.05E+02	3.65E+05	3.00E+02
145	HH.m281.081225		published data	2.94E+09	5.76E+04	5.57E+07	1.38E+03	3.86E+02	1.15E+05	3.00E+02
146	HH.192.090930		published data	4.73E+09	9.57E+04	8.46E+07	1.15E+03	3.84E+02	6.94E+04	3.00E+02
147	HQ.m125.110820		published data	4.74E+09	6.43E+03	1.03E+07	3.57E+03	5.32E+02	1.08E+05	3.00E+02
148	HQ.m34.110906		published data	7.56E+09	6.82E+03	1.50E+07	9.40E+03	5.90E+02	1.75E+05	3.00E+02
149	HQ.m55.111105		published data	2.49E+09	4.65E+04	3.63E+07	8.19E+02	3.81E+02	1.42E+05	3.00E+02
150	HQ.47.130816		published data	9.40E+09	3.51E+05	3.17E+08	1.16E+03	4.01E+02	1.75E+05	3.00E+02
151	HQ.59.130113		published data	4.92E+09	2.82E+04	2.39E+07	1.09E+03	3.67E+02	1.28E+05	3.00E+02
152	JJ.m100.110929		published data	4.29E+09	1.94E+04	2.51E+07	4.26E+03	4.14E+02	2.32E+05	3.00E+02
153	JJ.m88.110820		published data	4.65E+09	1.33E+04	2.14E+07	4.90E+03	4.90E+02	2.40E+05	3.00E+02

154	JJ.m95.110911		published data	7.88E+09	1.69E+04	3.09E+07	4.12E+03	6.09E+02	1.13E+05	3.00E+02
155	JJ.234.130208		published data	4.88E+09	3.74E+04	2.91E+07	9.32E+02	3.61E+02	5.62E+04	3.00E+02
156	JJ.257.130514		published data	5.50E+09	1.03E+05	8.98E+07	1.08E+03	3.95E+02	5.27E+04	3.00E+02
157	NN.m221.080829		published data	2.65E+09	3.33E+04	3.23E+07	1.81E+03	3.78E+02	1.28E+05	3.00E+02
158	NN.m228.081002		published data	4.99E+09	2.70E+04	2.80E+07	1.77E+03	3.95E+02	1.73E+05	3.00E+02
159	NN.144.090425		published data	7.39E+09	6.29E+04	7.17E+07	2.09E+03	4.14E+02	3.05E+05	3.00E+02
160	NN.m238.081204		published data	3.01E+09	4.68E+04	4.91E+07	2.19E+03	3.83E+02	3.58E+05	3.00E+02
161	NN.149.091002		published data	7.39E+09	5.29E+04	4.46E+07	1.02E+03	3.89E+02	4.59E+04	3.00E+02
162	QF.m322.080927		published data	5.01E+09	3.92E+03	1.21E+07	2.54E+04	7.57E+02	1.73E+05	3.00E+02
163	QF.131.090529		published data	5.16E+09	3.94E+04	3.73E+07	1.31E+03	3.98E+02	1.37E+05	3.00E+02
164	QF.m330.081218		published data	4.10E+09	5.71E+04	6.39E+07	2.68E+03	3.88E+02	3.76E+05	3.00E+02
165	QF.m333.090121		published data	4.04E+09	6.11E+04	7.06E+07	2.39E+03	4.05E+02	4.95E+05	3.00E+02

166	QF.135.091001		published data	6.93E+09	9.41E+04	8.50E+07	1.15E+03	3.93E+02	2.43E+05	3.00E+02
167	QQ.m135.110814		published data	4.88E+09	3.69E+03	1.35E+07	4.00E+04	1.02E+03	3.39E+05	3.00E+02
168	QQ.m152.111015		published data	6.50E+09	2.28E+04	2.60E+07	2.35E+03	4.07E+02	1.67E+05	3.00E+02
169	QQ.102.130620		published data	5.05E+09	2.11E+05	1.80E+08	1.07E+03	3.93E+02	6.04E+04	3.00E+02
170	QQ.98.130411		published data	4.80E+09	6.36E+04	5.97E+07	1.28E+03	3.95E+02	5.52E+04	3.00E+02
171	SY.m195.080827		published data	4.90E+09	2.12E+04	3.31E+07	4.80E+03	4.70E+02	2.96E+05	3.00E+02
172	SY.m202.081002		published data	1.67E+09	3.29E+04	3.46E+07	2.16E+03	3.88E+02	2.06E+05	3.00E+02
173	SY.m210.081125		published data	1.41E+09	4.85E+04	4.02E+07	9.30E+02	3.64E+02	3.35E+05	3.00E+02
174	SY.167.091121		published data	7.13E+09	5.05E+04	6.95E+07	3.88E+03	4.49E+02	2.48E+05	3.00E+02
175	YL.m252.080918		published data	5.44E+09	9.92E+03	2.35E+07	1.87E+04	6.05E+02	2.68E+05	3.00E+02
176	YL.m265.090109		published data	5.05E+09	3.65E+04	5.13E+07	3.97E+03	4.40E+02	3.76E+05	3.00E+02
177	YL.m264.081212		published data	4.45E+09	2.99E+04	4.12E+07	3.80E+03	4.42E+02	1.32E+05	3.00E+02

178	YL.173.090701		published data	5.30E+09	3.43E+04	7.01E+07	9.13E+03	5.54E+02	3.82E+05	3.00E+02
179	YL.178.091015		published data	6.98E+09	7.34E+04	8.34E+07	2.04E+03	4.20E+02	1.40E+05	3.00E+02
180	ZZ.m173.110828		published data	4.92E+09	6.87E+03	2.22E+07	2.04E+04	9.83E+02	3.43E+05	3.00E+02
181	ZZ.m183.110929		published data	4.42E+09	4.25E+04	3.25E+07	8.64E+02	3.68E+02	1.08E+05	3.00E+02
182	ZZ.m188.111015		published data	4.17E+09	2.21E+04	2.08E+07	1.39E+03	3.88E+02	1.08E+05	3.00E+02
183	ZZ.109.130808		published data	5.25E+09	4.98E+04	3.51E+07	7.74E+02	3.65E+02	4.46E+04	3.00E+02
184	FY1.27		published data	5.49E+09	1.97E+05	9.50E+07	4.25E+02	3.17E+02	9.65E+04	3.00E+02
185	DL1		published data	5.30E+09	1.19E+05	6.61E+07	5.00E+02	3.24E+02	8.88E+04	3.00E+02
186	WG		published data	5.16E+09	2.28E+05	1.11E+08	4.46E+02	3.20E+02	8.87E+04	3.00E+02
187	GZ1.9.2		published data	5.56E+09	9.53E+04	7.79E+07	1.02E+03	3.50E+02	3.43E+05	3.00E+02
188	ZM2.1.27		published data	5.90E+09	4.68E+04	4.01E+07	1.06E+03	3.60E+02	7.60E+05	3.00E+02
189	HH		published data	4.72E+09	1.75E+05	9.17E+07	4.69E+02	3.22E+02	1.52E+05	3.00E+02

Table S6 All the 144 MGS.

MGS_ID	Genes	kindom	phylum	class	order	family	genus	species
CAG00001	15966	*Bacteria*	*Proteobacteria*	*Gammaproteobacteria*	*Enterobacteriales*	*Enterobacteriaceae*		
CAG00007	7944	*Bacteria*	*Proteobacteria*	*Alphaproteobacteria*	*Rhizobiales*	*Rhizobiaceae*	*Rhizobium*	
CAG00008	6970	*Bacteria*	*Proteobacteria*	*Gammaproteobacteria*	*Enterobacteriales*	*Enterobacteriaceae*	*Providencia*	*Providencia_alcalifaciens*
CAG00009	6363	*Bacteria*	*Firmicutes*	*Bacilli*	*Lactobacillales*	*Leuconostocaceae*		
CAG00010	5741	*Bacteria*	*Proteobacteria*	*Gammaproteobacteria*	*Enterobacteriales*	*Enterobacteriaceae*	*Kluyvera*	
CAG00011	5735	*Bacteria*	*Proteobacteria*	*Gammaproteobacteria*	*Enterobacteriales*	*Enterobacteriaceae*	*Hafnia*	*Hafnia_alvei*
CAG00012	5337	*Bacteria*	*Firmicutes*	*Bacilli*	*Lactobacillales*	*Streptococcaceae*	*Lactococcus*	*Lactococcus_raffinolactis*
CAG00013	5213	*Bacteria*	*Proteobacteria*	*Gammaproteobacteria*	*Enterobacteriales*	*Enterobacteriaceae*	*Pantoea*	*Pantoea_ananatis*
CAG00014	4932	*Bacteria*	*Proteobacteria*	*Gammaproteobacteria*	*Enterobacteriales*	*Enterobacteriaceae*		
CAG00015	4663	*Bacteria*	*Proteobacteria*	*Gammaproteobacteria*	*Enterobacteriales*	*Enterobacteriaceae*	*Klebsiella*	

CAG00016	4529	*Bacteria*	*Firmicutes*					
CAG00017	4502	*Bacteria*	*Proteobacteria*	*Gammaproteobacteria*	*Enterobacteriales*	*Enterobacteriaceae*	*Raoultella*	
CAG00018	4486	*Bacteria*	*Actinobacteria*	*Actinobacteria*	*Propionibacteriales*	*Propionibacteriaceae*	*Propionibacterium*	
CAG00021	4191	*Bacteria*	*Firmicutes*	*Erysipelotrichia*	*Erysipelotrichales*	*Erysipelotrichaceae*	*Turicibacter*	*Turicibacter_sanguinis*
CAG00023	3960	*Bacteria*	*Proteobacteria*	*Gammaproteobacteria*	*Enterobacteriales*	*Enterobacteriaceae*	*Citrobacter*	
CAG00024	3839	*Bacteria*						
CAG00026	3638	*Bacteria*	*Proteobacteria*	*Gammaproteobacteria*	*Enterobacteriales*	*Enterobacteriaceae*		
CAG00027	3632	*Bacteria*	*Proteobacteria*	*Gammaproteobacteria*	*Enterobacteriales*	*Enterobacteriaceae*	*Leclercia*	*Leclercia_adecarboxylata*
CAG00028	3451	*Bacteria*	*Proteobacteria*	*Gammaproteobacteria*	*Enterobacteriales*	*Enterobacteriaceae*	*Kluyvera*	*Kluyvera_ascorbata*
CAG00034	3178	*Bacteria*	*Firmicutes*	*Clostridia*	*Clostridiales*	*Clostridiaceae*	*Clostridium*	
CAG00035	3177	*Bacteria*	*Proteobacteria*	*Gammaproteobacteria*	*Enterobacteriales*	*Enterobacteriaceae*	*Enterobacter*	
CAG00036	3176	*Bacteria*	*Proteobacteria*	*Gammaproteobacteria*	*Pseudomonadales*	*Pseudomonadaceae*	*Pseudomonas*	*Pseudomonas_fragi*

CAG00037	3170	*Bacteria*	*Firmicutes*	*Bacilli*	*Lactobacillales*	*Leuconostocaceae*	*Leuconostoc*	
CAG00039	3010	*Bacteria*	*Proteobacteria*	*Gammaproteobacteria*	*Enterobacteriales*	*Enterobacteriaceae*		
CAG00040	2969	*Bacteria*	*Proteobacteria*	*Gammaproteobacteria*	*Enterobacteriales*	*Enterobacteriaceae*	*Morganella*	*Morganella_morganii*
CAG00041	2944	*Bacteria*	*Proteobacteria*	*Gammaproteobacteria*	*Pseudomonadales*	*Pseudomonadaceae*	*Pseudomonas*	*Pseudomonas_helleri*
CAG00042	2934	*Bacteria*	*Firmicutes*	*Bacilli*	*Lactobacillales*	*Enterococcaceae*	*Enterococcus*	
CAG00043	2835	*Bacteria*	*Firmicutes*	*Bacilli*	*Lactobacillales*	*Streptococcaceae*	*Streptococcus*	*Streptococcus_orisratti*
CAG00044	2822	*Bacteria*	*Firmicutes*	*Erysipelotrichia*	*Erysipelotrichales*	*Erysipelotrichaceae*	*Turicibacter*	
CAG00045	2809	*Bacteria*	*Firmicutes*	*Erysipelotrichia*	*Erysipelotrichales*	*Erysipelotrichaceae*	*Turicibacter*	*unclassified_g__Turicibacter*
CAG00046	2788	*Bacteria*	*Proteobacteria*	*Gammaproteobacteria*	*Enterobacteriales*	*Enterobacteriaceae*	*Providencia*	*Providencia_rettgeri*
CAG00047	2750	*Bacteria*	*Proteobacteria*	*Gammaproteobacteria*	*Enterobacteriales*	*Enterobacteriaceae*		
CAG00048	2750	*Bacteria*	*Proteobacteria*	*Gammaproteobacteria*	*Enterobacteriales*	*Enterobacteriaceae*	*Cedecea*	*Cedecea_davisae*
CAG00049	2738	*Bacteria*	*Proteobacteria*	*Gammaproteobacteria*	*Enterobacteriales*	*Enterobacteriaceae*	*Moellerella*	*Moellerella_wisconsensis*

CAG00050	2667	*Bacteria*	*Proteobacteria*	*Gammaproteobacteria*	*Pseudomonadales*	*Pseudomonadaceae*	*Pseudomonas*	
CAG00051	2662	*Bacteria*	*Proteobacteria*	*Gammaproteobacteria*	*Enterobacteriales*	*Enterobacteriaceae*	*Yersinia*	
CAG00052	2597	*Bacteria*	*Proteobacteria*	*Gammaproteobacteria*	*Enterobacteriales*	*Enterobacteriaceae*	*Escherichia*	
CAG00053	2590	*Bacteria*	*Firmicutes*	*Clostridia*	*Clostridiales*	*Clostridiaceae*	*Clostridium*	
CAG00054	2570	*Bacteria*	*Firmicutes*	*Clostridia*	*Clostridiales*	*Lachnospiraceae*	*Cellulosilyticum*	*Cellulosilyticum_lentocellum*
CAG00055	2564	*Bacteria*	*Firmicutes*	*Clostridia*	*Clostridiales*	*Clostridiaceae*	*Clostridium*	*Clostridium_sp._JCC*
CAG00058	2530	*Bacteria*	*Proteobacteria*	*Gammaproteobacteria*	*Pseudomonadales*	*Pseudomonadaceae*	*Pseudomonas*	*Pseudomonas_fragi*
CAG00061	2462	*Bacteria*	*Firmicutes*	*Clostridia*	*Clostridiales*	*Peptostreptococcaceae*	*Terrisporobacter*	*Terrisporobacter_othiniensis*
CAG00062	2429	*Bacteria*	*Firmicutes*	*Clostridia*	*Clostridiales*	*Clostridiaceae*	*Clostridium*	
CAG00063	2423	*Bacteria*	*Firmicutes*	*Clostridia*	*Clostridiales*	*Clostridiaceae*	*Clostridium*	*Clostridium_sp._7_2_43FAA*
CAG00065	2404	*Bacteria*	*Firmicutes*	*Clostridia*	*Clostridiales*	*Clostridiaceae*	*Clostridium*	
CAG00070	2337	*Bacteria*	*Firmicutes*	*Bacilli*	*Lactobacillales*	*Enterococcaceae*	*Enterococcus*	*Enterococcus_casseliflavus*

CAG00071	2326	*Bacteria*	*Firmicutes*	*Bacilli*	*Lactobacillales*	*Lactobacillaceae*	*Lactobacillus*	*Lactobacillus_plantarum*
CAG00072	2276	*Bacteria*	*Proteobacteria*	*Gammaproteobacteria*	*Enterobacteriales*	*Enterobacteriaceae*	*Rahnella*	
CAG00074	2207	*Bacteria*	*Proteobacteria*	*Gammaproteobacteria*	*Pasteurellales*	*Pasteurellaceae*	*Actinobacillus*	*Actinobacillus_succinogenes*
CAG00075	2171	*Bacteria*	*Firmicutes*	*Bacilli*	*Lactobacillales*	*Enterococcaceae*	*Enterococcus*	
CAG00076	2170	*Bacteria*	*Firmicutes*	*Clostridia*	*Clostridiales*	*Clostridiaceae*	*Clostridium*	
CAG00077	2159	*Bacteria*	*Proteobacteria*	*Gammaproteobacteria*	*Enterobacteriales*	*Enterobacteriaceae*		
CAG00079	2140	*Bacteria*	*Proteobacteria*	*Gammaproteobacteria*	*Pseudomonadales*	*Pseudomonadaceae*	*Pseudomonas*	*Pseudomonas_helleri*
CAG00080	2130	*Bacteria*						
CAG00081	2128	*Bacteria*	*Firmicutes*	*Bacilli*	*Lactobacillales*	*Streptococcaceae*	*Streptococcus*	*Streptococcus_criceti*
CAG00082	2121	*Bacteria*	*Firmicutes*	*Bacilli*	*Bacillales*	*Bacillaceae*	*Bacillus*	
CAG00083	2071	*Bacteria*	*Firmicutes*	*Bacilli*	*Lactobacillales*	*Enterococcaceae*	*Enterococcus*	*unclassified_g__Enterococcus*
CAG00085	2028	*Bacteria*	*Firmicutes*	*Bacilli*	*Lactobacillales*	*Streptococcaceae*	*Lactococcus*	*Lactococcus_lactis*

CAG00089	1952	*Bacteria*	*Firmicutes*	*Bacilli*	*Lactobacillales*	*Leuconostocaceae*	*Leuconostoc*	*Leuconostoc_lactis*
CAG00090	1940	*Bacteria*	*Proteobacteria*	*Epsilonproteobacteria*	*Campylobacterales*	*Helicobacteraceae*	*Helicobacter*	
CAG00091	1936	*Bacteria*	*Proteobacteria*	*Epsilonproteobacteria*	*Campylobacterales*	*Campylobacteraceae*	*Campylobacter*	*Campylobacter_jejuni*
CAG00093	1916	*Bacteria*	*Proteobacteria*	*Gammaproteobacteria*	*Pseudomonadales*	*Pseudomonadaceae*	*Pseudomonas*	
CAG00096	1861	*Bacteria*	*Firmicutes*	*Bacilli*	*Lactobacillales*	*Lactobacillaceae*	*Lactobacillus*	*Lactobacillus_reuteri*
CAG00098	1831	*Bacteria*	*Firmicutes*	*Clostridia*	*Clostridiales*	*Clostridiaceae*	*Clostridium*	
CAG00100	1830	*Bacteria*	*Firmicutes*	*Bacilli*	*Lactobacillales*	*Streptococcaceae*	*Lactococcus*	*Lactococcus_garvieae*
CAG00101	1828	*Bacteria*	*Proteobacteria*	*Gammaproteobacteria*	*Enterobacteriales*	*Enterobacteriaceae*	*Escherichia*	*Escherichia_coli*
CAG00102	1812	*Bacteria*	*Proteobacteria*	*Gammaproteobacteria*	*Pseudomonadales*	*Pseudomonadaceae*	*Pseudomonas*	*Pseudomonas_sp._CBZ-4*
CAG00103	1805	*Bacteria*	*Firmicutes*	*Bacilli*	*Lactobacillales*	*Enterococcaceae*	*Enterococcus*	*Enterococcus_sulfureus*
CAG00105	1741	*Bacteria*	*Firmicutes*	*Bacilli*	*Lactobacillales*	*Streptococcaceae*	*Streptococcus*	*unclassified_g__Streptococcus*
CAG00106	1738	*Bacteria*	*Proteobacteria*	*Gammaproteobacteria*	*Enterobacteriales*	*Enterobacteriaceae*		

CAG00107	1736	*Bacteria*	*Firmicutes*	*Bacilli*	*Lactobacillales*	*Streptococcaceae*	*Streptococcus*	
CAG00108	1709	*Bacteria*	*Firmicutes*	*Bacilli*	*Lactobacillales*	*Streptococcaceae*	*Streptococcus*	*Streptococcus_caballi*
CAG00113	1668	*Bacteria*	*Proteobacteria*	*Gammaproteobacteria*	*Enterobacteriales*	*Enterobacteriaceae*	*Providencia*	*Providencia_alcalifaciens*
CAG00114	1660	*Bacteria*	*Firmicutes*	*Clostridia*	*Clostridiales*	*Clostridiaceae*	*Clostridium*	
CAG00115	1656	*Bacteria*	*Firmicutes*	*Bacilli*	*Lactobacillales*	*Streptococcaceae*	*Lactococcus*	*Lactococcus_lactis*
CAG00116	1642	*Bacteria*	*Proteobacteria*	*Gammaproteobacteria*	*Enterobacteriales*	*Enterobacteriaceae*		
CAG00119	1636	*Bacteria*	*Proteobacteria*	*Alphaproteobacteria*	*Rhizobiales*	*Bradyrhizobiaceae*	*Afipia*	*unclassified_g__Afipia*
CAG00120	1634	*Bacteria*	*Proteobacteria*	*Gammaproteobacteria*	*Enterobacteriales*	*Enterobacteriaceae*	*Enterobacter*	
CAG00122	1618	*Bacteria*	*Firmicutes*	*Bacilli*	*Lactobacillales*	*Leuconostocaceae*	*Weissella*	*Weissella_confusa*
CAG00123	1617	*Bacteria*	*Proteobacteria*	*Gammaproteobacteria*	*Enterobacteriales*	*Enterobacteriaceae*		
CAG00125	1607	*Bacteria*	*Proteobacteria*	*Gammaproteobacteria*	*Enterobacteriales*	*Enterobacteriaceae*	*Hafnia*	*Hafnia_alvei*
CAG00126	1563	*Bacteria*	*Proteobacteria*	*Gammaproteobacteria*	*Enterobacteriales*	*Enterobacteriaceae*		

CAG00127	1562	*Bacteria*	*Firmicutes*	*Clostridia*	*Clostridiales*	*Clostridiaceae*	*Clostridium*	
CAG00130	1516	*Bacteria*	*Firmicutes*	*Bacilli*	*Lactobacillales*	*Lactobacillaceae*	*Lactobacillus*	*Lactobacillus_mucosae*
CAG00131	1515	*Bacteria*	*Proteobacteria*	*Alphaproteobacteria*	*Rhizobiales*			
CAG00132	1506	*Bacteria*	*Firmicutes*	*Clostridia*	*Clostridiales*	*Clostridiaceae*	*Clostridium*	*Clostridium_sp._CL-6*
CAG00133	1467	*Fungi*	*Ascomycota*	*Dothideomycetes*	*Pleosporales*	*Phaeosphaeriaceae*	*Parastagonospora*	*Parastagonospora_nodorum*
CAG00134	1457	*Bacteria*	*Firmicutes*	*Clostridia*	*Clostridiales*	*Clostridiaceae*	*Clostridium*	
CAG00135	1448	*Bacteria*	*Proteobacteria*	*Gammaproteobacteria*	*Pseudomonadales*	*Pseudomonadaceae*	*Pseudomonas*	
CAG00136	1445	*Bacteria*	*Proteobacteria*	*Gammaproteobacteria*	*Pseudomonadales*	*Pseudomonadaceae*	*Pseudomonas*	*Pseudomonas_fragi*
CAG00137	1444	*Bacteria*	*Proteobacteria*	*Gammaproteobacteria*	*Enterobacteriales*	*Enterobacteriaceae*	*Enterobacter*	
CAG00139	1422	*Bacteria*	*Proteobacteria*	*Gammaproteobacteria*	*Pseudomonadales*	*Pseudomonadaceae*	*Pseudomonas*	
CAG00142	1388	*Bacteria*	*Firmicutes*	*Bacilli*	*Lactobacillales*	*Streptococcaceae*	*Streptococcus*	*Streptococcus_sobrinus*
CAG00143	1370	*Bacteria*	*Actinobacteria*	*Actinobacteria*	*Micrococcales*	*Microbacteriaceae*	*Microbacterium*	

CAG00144	1358	*Bacteria*	*Firmicutes*	*Clostridia*	*Clostridiales*	*Lachnospiraceae*	*Cellulosilyticum*	*Cellulosilyticum_lentocellum*
CAG00145	1338	*Bacteria*	*Firmicutes*	*Bacilli*	*Lactobacillales*	*Streptococcaceae*	*Streptococcus*	*Streptococcus_ratti*
CAG00147	1323	*Bacteria*	*Proteobacteria*	*Gammaproteobacteria*	*Pseudomonadales*	*Pseudomonadaceae*	*Pseudomonas*	
CAG00148	1321	*Bacteria*	*Proteobacteria*	*Gammaproteobacteria*	*Enterobacteriales*	*Enterobacteriaceae*	*Escherichia*	*Escherichia_fergusonii*
CAG00149	1320	*Bacteria*	*Proteobacteria*	*Gammaproteobacteria*	*Enterobacteriales*	*Enterobacteriaceae*		
CAG00150	1316	*Bacteria*	*Proteobacteria*	*Gammaproteobacteria*	*Pseudomonadales*	*Moraxellaceae*	*Acinetobacter*	*Acinetobacter_rudis*
CAG00151	1313	*Bacteria*	*Proteobacteria*	*Gammaproteobacteria*	*Pseudomonadales*	*Pseudomonadaceae*	*Pseudomonas*	*Pseudomonas_deceptionensis*
CAG00153	1262	*Bacteria*	*Firmicutes*	*Bacilli*	*Lactobacillales*	*Streptococcaceae*	*Streptococcus*	
CAG00154	1227	*Bacteria*	*Firmicutes*	*Negativicutes*	*Selenomonadales*	*Veillonellaceae*	*Veillonella*	*Veillonella_sp._AS16*
CAG00156	1212	*Bacteria*	*Proteobacteria*	*Gammaproteobacteria*	*Pseudomonadales*	*Pseudomonadaceae*	*Pseudomonas*	
CAG00157	1211	*Bacteria*	*Firmicutes*	*Bacilli*	*Bacillales*	*Bacillaceae*	*Lysinibacillus*	*Lysinibacillus_contaminans*
CAG00158	1196	*Bacteria*	*Firmicutes*	*Bacilli*	*Lactobacillales*	*Enterococcaceae*	*Enterococcus*	*Enterococcus_raffinosus*

CAG00160	1178	*Bacteria*	*Proteobacteria*	*Gammaproteobacteria*	*Enterobacteriales*	*Enterobacteriaceae*		
CAG00161	1167	*Bacteria*	*Firmicutes*	*Bacilli*	*Lactobacillales*	*Enterococcaceae*	*Enterococcus*	*unclassified_g__Enterococcus*
CAG00162	1161	*Bacteria*	*Proteobacteria*	*Gammaproteobacteria*	*Enterobacteriales*	*Enterobacteriaceae*	*Escherichia*	*Escherichia_coli*
CAG00163	1160	*Bacteria*	*Firmicutes*	*Bacilli*	*Lactobacillales*	*Streptococcaceae*	*Streptococcus*	*Streptococcus_ferus*
CAG00164	1154	*Bacteria*	*Proteobacteria*	*Gammaproteobacteria*				
CAG00165	1146	*Bacteria*	*Proteobacteria*	*Gammaproteobacteria*	*Enterobacteriales*	*Enterobacteriaceae*	*Escherichia*	*Escherichia_coli*
CAG00168	1113	*Bacteria*	*Firmicutes*	*Erysipelotrichia*	*Erysipelotrichales*	*Erysipelotrichaceae*	*Turicibacter*	*Turicibacter_sanguinis*
CAG00169	1109	*Bacteria*	*Proteobacteria*	*Gammaproteobacteria*	*Aeromonadales*	*Aeromonadaceae*	*Aeromonas*	*Aeromonas_hydrophila*
CAG00171	1069	*Bacteria*	*Proteobacteria*	*Gammaproteobacteria*	*Enterobacteriales*	*Enterobacteriaceae*	*Cedecea*	*Cedecea_davisae*
CAG00173	1040	*Bacteria*	*Firmicutes*	*Bacilli*	*Lactobacillales*	*Streptococcaceae*	*Lactococcus*	*Lactococcus_lactis*
CAG00174	1031	*Bacteria*	*Proteobacteria*	*Gammaproteobacteria*	*Enterobacteriales*	*Enterobacteriaceae*	*Serratia*	
CAG00175	1026	*Bacteria*	*Proteobacteria*	*Gammaproteobacteria*	*Enterobacteriales*	*Enterobacteriaceae*	*Raoultella*	*Raoultella_ornithinolytica*

CAG00177	985	*Bacteria*	*Firmicutes*	*Clostridia*	*Clostridiales*	*Clostridiaceae*	*Clostridium*	
CAG00178	977	*Bacteria*	*Firmicutes*	*Bacilli*	*Lactobacillales*	*Leuconostocaceae*	*Weissella*	*Weissella_cibaria*
CAG00180	967	*Bacteria*	*Firmicutes*	*Bacilli*	*Lactobacillales*	*Lactobacillaceae*	*Lactobacillus*	*Lactobacillus_johnsonii*
CAG00182	954	*Bacteria*	*Firmicutes*	*Clostridia*	*Clostridiales*	*Clostridiaceae*	*Clostridium*	*Clostridium_sp._CAG:221*
CAG00183	954	*Bacteria*	*Firmicutes*	*Clostridia*	*Clostridiales*	*Clostridiaceae*	*Clostridium*	
CAG00185	951	*Bacteria*	*Proteobacteria*	*Gammaproteobacteria*	*Enterobacteriales*	*Enterobacteriaceae*		
CAG00186	943	*Bacteria*	*Proteobacteria*	*Gammaproteobacteria*	*Enterobacteriales*	*Enterobacteriaceae*	*Citrobacter*	
CAG00188	928	*Bacteria*	*Firmicutes*	*Bacilli*	*Bacillales*	*Bacillaceae*	*Lysinibacillus*	
CAG00190	913	*Bacteria*	*Proteobacteria*	*Gammaproteobacteria*	*Enterobacteriales*	*Enterobacteriaceae*		
CAG00191	912	*Bacteria*	*Proteobacteria*	*Gammaproteobacteria*	*Enterobacteriales*	*Enterobacteriaceae*	*Providencia*	
CAG00192	904	*Bacteria*	*Firmicutes*	*Clostridia*	*Clostridiales*	*Clostridiaceae*	*Clostridium*	
CAG00193	897	*Bacteria*	*Firmicutes*	*Clostridia*	*Clostridiales*	*Clostridiaceae*	*Clostridium*	*Clostridium_sp._ND2*

CAG00195	872	*Bacteria*	*Proteobacteria*	*Gammaproteobacteria*	*Pseudomonadales*	*Pseudomonadaceae*	*Pseudomonas*	
CAG00197	842	*Bacteria*	*Proteobacteria*	*Gammaproteobacteria*	*Enterobacteriales*	*Enterobacteriaceae*	*Enterobacter*	
CAG00201	822	*Bacteria*	*Firmicutes*	*Bacilli*	*Lactobacillales*	*Lactobacillaceae*	*Lactobacillus*	
CAG00202	814	*Bacteria*	*Proteobacteria*	*Gammaproteobacteria*	*Enterobacteriales*	*Enterobacteriaceae*		
CAG00203	814	*Bacteria*	*Firmicutes*	*Clostridia*	*Clostridiales*	*Clostridiaceae*	*Clostridium*	*Clostridium_sp._Ade.TY*
CAG00206	790	*Bacteria*	*Proteobacteria*	*Gammaproteobacteria*	*Enterobacteriales*	*Enterobacteriaceae*		
CAG00207	790	*Bacteria*	*Proteobacteria*	*Gammaproteobacteria*	*Enterobacteriales*	*Enterobacteriaceae*	*Escherichia*	
CAG00208	788	*Bacteria*	*Proteobacteria*	*Gammaproteobacteria*	*Enterobacteriales*	*Enterobacteriaceae*	*Escherichia*	*Escherichia_coli*
CAG00215	749	*Bacteria*	*Proteobacteria*	*Gammaproteobacteria*	*Enterobacteriales*	*Enterobacteriaceae*	*Enterobacter*	
CAG00216	747	*Bacteria*	*Proteobacteria*	*Gammaproteobacteria*	*Enterobacteriales*	*Enterobacteriaceae*	*Citrobacter*	*Citrobacter_freundii*
CAG00217	745	*Bacteria*	*Proteobacteria*	*Gammaproteobacteria*	*Enterobacteriales*	*Enterobacteriaceae*	*Citrobacter*	*Citrobacter_pasteurii*
CAG00218	745	*Bacteria*	*Proteobacteria*	*Gammaproteobacteria*	*Enterobacteriales*	*Enterobacteriaceae*	*Morganella*	*Morganella_morganii*

CAG00220	736	*Bacteria*	*Firmicutes*	*Clostridia*	*Clostridiales*	*Clostridiaceae*	*Clostridium*	
CAG00225	715	*Bacteria*						

Table S7 All the 112 nonflavonoid compounds (NFCs) in feces.

NFC_no.	Compound name	Class	HMDB
1	COAMARIC ACID	phenylpropanoid	HMDB0041592
2	Indoleacetic acid	Indoles	HMDB0000197
3	Salicyluric acid	phenylpropanoid	HMDB0000840
4	Glutaric acid	Organic Acids	HMDB0000661
5	N_acetyltryptophan	Amino Acids	HMDB0013713
6	Cinnamic acid and trans_Cinnamic acid	phenylpropanoid	HMDB0000567/ HMDB0000930
7	2_Phenylpropionate	phenylpropanoid	HMDB0011743
8	Hydrocinnamic acid	phenylpropanoid	HMDB0000764
9	Indoleacrylic acid	Indoles	HMDB0000734
10	3_Indolepropionic acid	Indoles	HMDB0002302
11	4_Hydroxyphenylpyruvic acid	benzenoids	HMDB0000707
12	3_Methyl_2_oxovaleric acid	Organic Acids	HMDB0000491
13	Glycine	Amino Acids	HMDB0000123
14	Glycolic acid	Organic Acids	HMDB0000115
15	3_Aminoisobutanoic acid	Amino Acids	HMDB0003911
16	Glyceric acid	Sugar Acids	HMDB0000139
17	L_Proline	Amino Acids	HMDB0000162
18	L_Pipecolic acid	Amino Acids	HMDB0000716

19	p_Cresol sulfate	Organic Acids	HMDB0011635
20	L_Valine	Amino Acids	HMDB0000883
21	Pyroglutamic acid	Amino Acids	HMDB0000267
22	3_Hydroxybutyric acid	Organic Acids	HMDB0000357
23	2_Hydroxybutyric acid	Fatty Acids	HMDB0000008
24	3_Hydroxyisovaleric acid	Fatty Acids	HMDB0000754
25	L_Isoleucine and L_Leucine	Amino Acids	HMDB0000172/HMDB0000687
26	C3 0	Fatty Acids	HMDB0000237
27	Melatonin	Amino Acids	HMDB0001389
28	3_ 3_Hydroxyphenyl _3_hydroxypropanoic acid	phenylpropanoid	HMDB0002643
29	p_Hydroxyphenylacetic acid	Phenols	HMDB0000020
30	L_Tryptophan	Amino Acids	HMDB0000929
31	C4 0	Fatty Acids	HMDB0000039
32	Vanillic acid	Fatty Acids	HMDB0000484
33	Malonic acid	Fatty Acids	HMDB0000691
34	Tartaric acid	Sugar acids	HMDB0000956
35	L_3_Phenyllactic acid and Phenyllactic acid	phenylpropanoid	HMDB0000779
36	Oxalic acid	Organic Acids	HMDB0002329
37	Citraconic acid	Fatty Acids	HMDB0000634
38	Methylsuccinic acid	Fatty Acids	HMDB0001844

39	Adipic acid	Fatty Acids	HMDB0000448
40	L_Lysine	Amino Acids	HMDB0000182
41	cis_Aconitic acid	Tricarboxylic acids	HMDB0000072
42	Pimelic acid	Organic Acids	HMDB0000857
43	1 _2_Methylpentanoic acid	Fatty Acids	HMDB0031580
44	3_Methylpentanoic acid	Fatty Acids	HMDB0033774
45	L_Histidine	Amino Acids	HMDB0000177
46	C6 0	Fatty Acids	HMDB0000535
47	Suberic acid	Organic Acids	HMDB0000893
48	Citric acid	Tricarboxylic acids	HMDB0000094
49	Isocitric acid	Organic Acids	HMDB0000193
50	2_Methylhexanoic acid	Fatty Acids	HMDB0031594
51	C7 0	Fatty Acids	HMDB0000666
52	Oxoglutaric acid	Organic Acids	HMDB0000208
53	C8 0	Fatty Acids	HMDB0000482
54	Oxoadipic acid	Fatty Acids	HMDB0000225
55	C9 0	Fatty Acids	HMDB0000847
56	C10 0	Fatty Acids	HMDB0000511
57	5_Dodecenoic acid	Fatty Acids	NA

58	C12 0	Fatty Acids	HMDB0000638
59	C14 1 cis_9	Fatty Acids	HMDB0002000
60	Phenylethylamine	phenylpropanoid	HMDB0012275
61	C14 0	Fatty Acids	HMDB0000806
62	C15 0	Fatty Acids	HMDB0000826
63	C16 1 cis_9	Fatty Acids	HMDB0003229
64	C18 3 cis_9 12 15	Fatty Acids	NA
65	C18 2 cis_9 12	Fatty Acids	HMDB0000673
66	C20 4 cis_5 8 11 14	Fatty Acids	HMDB0001043
67	C20 3 cis_11 14 17 and C20 3 cis_8 11 14	Fatty Acids	HMDB0002925
68	C22 6 cis_4 7 10 13 16 19	Fatty Acids	HMDB0002183
69	C22 5 cis_4 7 10 13 16	Fatty Acids	HMDB0001976
70	Succinic acid	Fatty Acids	HMDB0000254
71	Citramalic acid	Fatty Acids	HMDB0000426
72	C16 0	Fatty Acids	HMDB0000220
73	C17 0	Fatty Acids	HMDB0002259
74	C18 0	Fatty Acids	HMDB0000827
75	C20 1 cis_11	Fatty Acids	HMDB0002231
76	C20 1 trans_11 and C20 1 cis_8	Fatty Acids	NA
77	C20 1 cis_5	Fatty Acids	NA
78	C22 3 cis_13 16 19	Fatty Acids	HMDB0002823

79	C20 0	Fatty Acids	HMDB0002212
80	C22 1 cis_13	Fatty Acids	HMDB0002068
81	C22 0	Fatty Acids	HMDB0000944
82	L_Serine	Amino Acids	HMDB0000187
83	Hydroxypropionic acid	Fatty Acids	HMDB0000700
84	Ornithine	Amino Acids	HMDB0000214
85	3_Methylindole	Indoles	HMDB0000466
86	Nicotinic acid	Organic Acids	HMDB0001488
87	2_Phenylglycine	Amino Acids	HMDB0002210
88	L_Tyrosine	Amino Acids	HMDB0000158
89	L_Asparagine	Amino Acids	HMDB0000168
90	L_Phenylalanine	Amino Acids	HMDB0000159
91	Hydroxyphenyllactic acid	phenylpropanoid	HMDB0000755
92	L_Aspartic acid	Amino Acids	HMDB0000191
93	Aminoadipic acid	Amino Acids	HMDB0000510
94	4_Hydroxybenzoic acid	Organic Acids	HMDB0000500
95	Ortho_Hydroxyphenylacetic acid	benzenoids	HMDB0000669
96	Hippuric acid	Organic Acids	HMDB0000714
97	L_Malic acid	Organic Acids	HMDB0000156
98	Ethylmethylacetic acid	Fatty Acids	HMDB0002176
99	Phenylacetic acid	Organic Acids	HMDB0000209

100	3_Hydroxyphenylacetic acid	Phenols	HMDB0000440
101	D_2_Hydroxyglutaric acid	Amino Acids	HMDB0000606
102	L_Methionine	Amino Acids	HMDB0000696
103	alpha_Hydroxyisobutyric acid	Fatty Acids	HMDB0000729
104	Dopamine	Phenols	HMDB0000073
105	Serotonin	Indoles	HMDB0000259
106	L_Glutamic acid	Amino Acids	HMDB0000148
107	Beta_Alanine	Amino Acids	HMDB0000056
108	L_Alanine	Amino Acids	HMDB0000161
109	C19 0	Fatty Acids	HMDB0000772
110	Gamma_Aminobutyric acid	Amino Acids	HMDB0000112
111	C24 1 CIS_15	Fatty Acids	HMDB0002368
112	C24 0	Fatty Acids	HMDB0002003
